# Structure, function and drug discovery of GPCR signaling

**DOI:** 10.1186/s43556-023-00156-w

**Published:** 2023-12-04

**Authors:** Lin Cheng, Fan Xia, Ziyan Li, Chenglong Shen, Zhiqian Yang, Hanlin Hou, Suyue Sun, Yuying Feng, Xihao Yong, Xiaowen Tian, Hongxi Qin, Wei Yan, Zhenhua Shao

**Affiliations:** 1 Division of Nephrology and Kidney Research Institute, State Key Laboratory of Biotherapy, West China Hospital, Sichuan University, Chengdu, 610041 Sichuan China; 2 Department of Otolaryngology Head and Neck Surgery, Sichuan Provincial People’s Hospital, University of Electronic Science and Technology of China, Chengdu, 610000 China; 3 Department of Neurosurgery, West China Hospital, Sichuan University, Chengdu, 610041 China; 4 Tianfu Jincheng Laboratory, Frontiers Medical Center, Chengdu, 610212 China

## Abstract

G protein-coupled receptors (GPCRs) are versatile and vital proteins involved in a wide array of physiological processes and responses, such as sensory perception (e.g., vision, taste, and smell), immune response, hormone regulation, and neurotransmission. Their diverse and essential roles in the body make them a significant focus for pharmaceutical research and drug development. Currently, approximately 35% of marketed drugs directly target GPCRs, underscoring their prominence as therapeutic targets. Recent advances in structural biology have substantially deepened our understanding of GPCR activation mechanisms and interactions with G-protein and arrestin signaling pathways. This review offers an in-depth exploration of both traditional and recent methods in GPCR structure analysis. It presents structure-based insights into ligand recognition and receptor activation mechanisms and delves deeper into the mechanisms of canonical and noncanonical signaling pathways downstream of GPCRs. Furthermore, it highlights recent advancements in GPCR-related drug discovery and development. Particular emphasis is placed on GPCR selective drugs, allosteric and biased signaling, polyphamarcology, and antibody drugs. Our goal is to provide researchers with a thorough and updated understanding of GPCR structure determination, signaling pathway investigation, and drug development. This foundation aims to propel forward-thinking therapeutic approaches that target GPCRs, drawing upon the latest insights into GPCR ligand selectivity, activation, and biased signaling mechanisms.

## Introduction

GPCRs, as the largest membrane protein superfamily, are categorized into five distinct subfamilies: the rhodopsin-like family (Class A), the secretin/adhesion family (Class B), the metabotropic family (Class C), the smoothened/frizzled family (Class F), and the taste2 family (Class T). GPCRs play a pivotal role in transducing signals from the extracellular environment to the intracellular environment, regulating a variety of physiological processes. The diversity of signals they relay encompasses odors, light, neurotransmitters, and kinins. All GPCRs adopt the classic seven-transmembrane helix structure, connected by three extracellular loops (ECL1-3) as well as three intracellular loops (ICL1-3). However, each GPCR subfamily exhibits unique structural characteristics and ligand-binding specificities, intricately linked to their physiological roles [1].

**Fig. 1:**
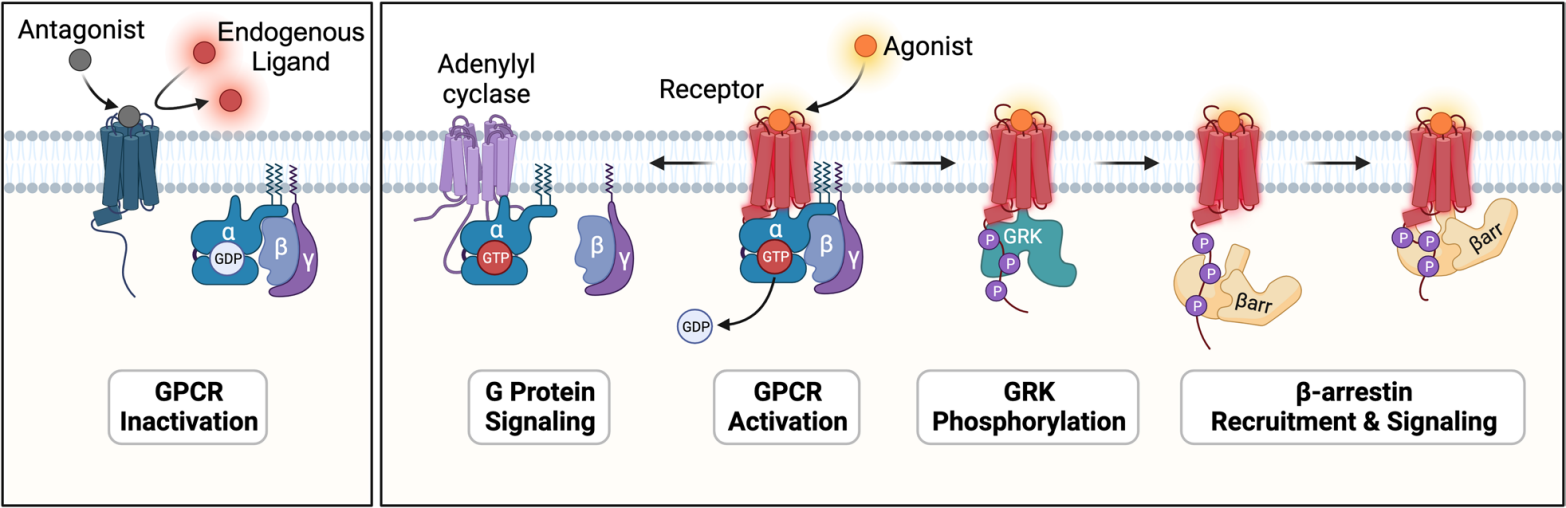
Scheme of ligand-mediated GPCR inactivation or activation. Agonists bind to GPCRs and trigger downstream G-protein or β-arrestin signaling. Antagonists occupy the agonism-associated pocket and prevent endogenous ligand binding

Over recent years, the landscape of GPCR investigation has been revolutionized by breakthroughs in structural biology, particularly with the advent of cryo-electron microscopy (cryo-EM) [2]. These advancements have shed light on the dynamic conformational changes of GPCRs, providing unprecedented insights into their inactivation or activation mechanisms and interactions with intracellular signaling transducers such as G-proteins and arrestins (Fig. 1) [3, 4]. Understanding the GPCR structure alterations, from inactive to active states, has been instrumental in deciphering the nuances of their signal transduction pathways and their implications on cellular responses.

Furthermore, a deepened comprehension of GPCR structural biology has significantly accelerated drug discovery endeavors [5]. With a precise grasp of ligand-receptor interactions and activation mechanisms, researchers have been empowered to rationally design and optimize drug candidates targeting GPCRs [6–8]. This has not only facilitated the identification of novel therapeutic agents but also enabled the refinement of existing drugs to enhance their efficacy, selectivity, and safety profiles [9].

Here, we aim to encapsulate the recent strides in GPCR structural elucidation, explore the implications of these findings on our understanding of GPCR-mediated signal transduction, and highlight the emerging opportunities in developing innovative GPCR-targeted drugs. Through a synthesis of current knowledge and prospects, we aspire to underscore the significance and potential of GPCRs in biomedical research and therapeutic development.

## Technology progress for GPCR structure determination

GPCRs are dynamic in the cell membrane and respond to different types of ligands, including agonists, antagonists or allosteric modulators. In turn, this characteristic governs various intracellular pathways. Understanding the structure of GPCRs is fundamental for understanding ligand recognition and the mechanism of receptor activation, which could accelerate drug discovery.

Nonetheless, the task of elucidating the GPCR structure has been challenging, primarily owing to their low expression levels and dynamic features. To overcome these challenges, X-ray crystallography and single-particle cryo-EM techniques were subsequently applied for GPCR structure determination. A total of 243 unique structures have been deposited in the Protein Data Bank (PDB), including 188 Class A GPCRs, 28 Class B GPCRs (20 for Class B1 and 8 for Class B2), 19 Class C GPCRs, 5 Class F GPCRs, and 1 Class T GPCR.

**Fig. 2:**
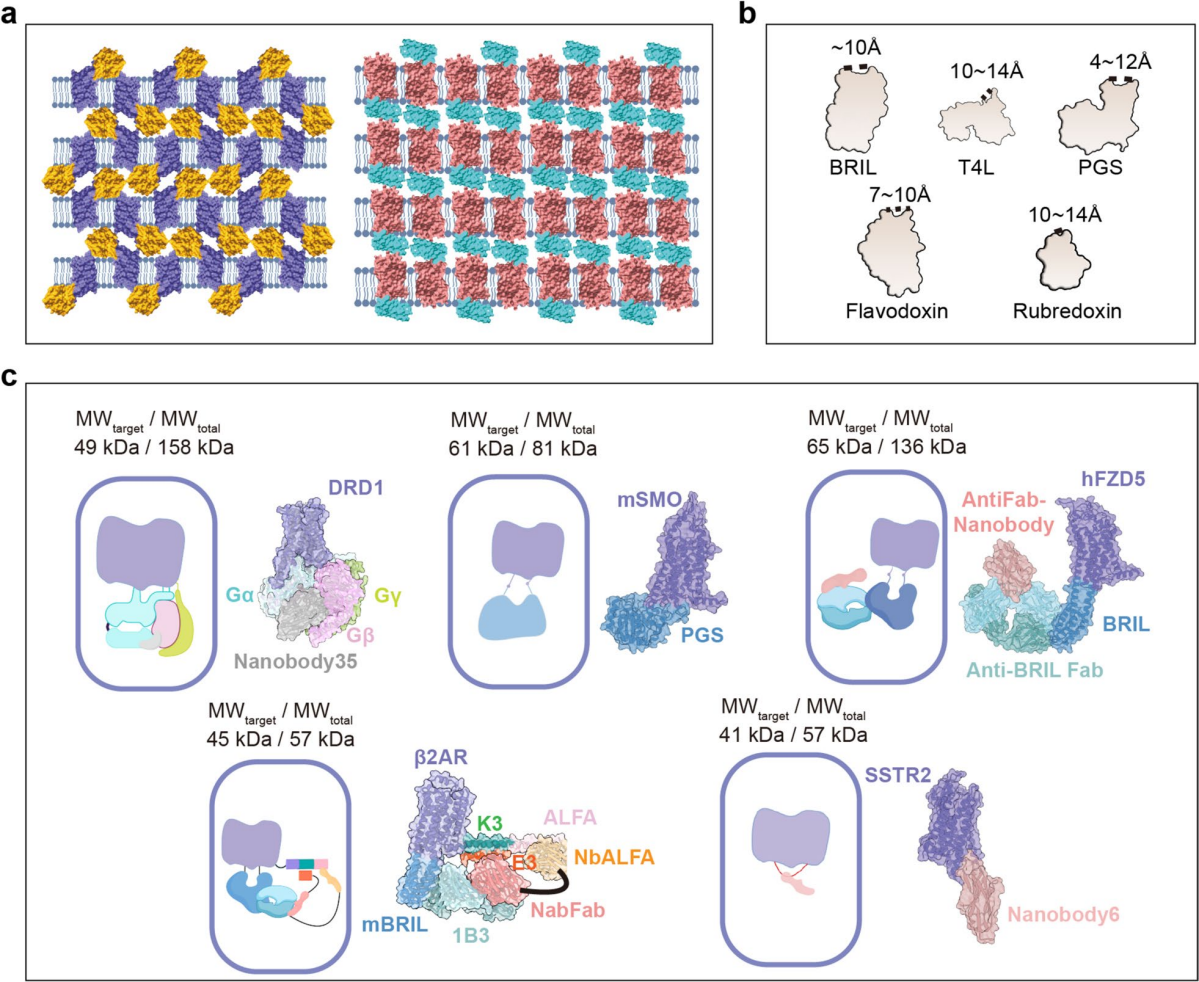
Techniques for GPCR structure determination. **a** Crystal packing for GPCRs in the presence of fusion proteins. GnRH1R-PGS (left, PDB: 7BR3), GnRH1R: purple, PGS: yellow. ghrelin-BRIL (right, PDB: 7F83), ghrelin: red, BRIL: green. **b** Schematic diagrams of fusion proteins. The dashed line shows the distances between the N-terminus and C-terminus of the fusion proteins. **c** Strategies for cryo-EM structure determination of GPCRs. The complex of D1R with G_s_ and Nb35 (PDB: 7CKZ). mSMO with PGS fusion protein (PDB: 8CXO). hFZD5 with BRIL fusion protein binding with anti-BRIL Fab and anti-Fab Nanobody (PDB: 6WW2). β2AR links ICL3 to engaging BRIL mBRIL and the C-terminus to the K3 helix with an ALFA tag. The complex involves an anti-BRIL Fab, along with a bivalent ‘glue’ molecule containing anti-Fab (NbFab) and anti-ALFA (NbALFA) (PDB: 8J7E and 8JJO). SSTR2 is bound to nanobody6 (PDB: 7UL5)

For X-ray crystallography, GPCRs are extracted from the cell membrane using synthetic detergents. This often reduces the hydrophilic surface essential for crystallization packing. To address these challenges, in 2007, Brian K. Kobilka’s team innovated by developing a fusion protein strategy combined with the lipid cubic phase (LCP) method (Fig. 2a, b) [10]. This approach facilitated both the stabilization and crystallization of GPCRs. Moreover, the introduction of specific mutations within the transmembrane domain was reported to enhance the thermostability of the receptor. A pivotal moment in GPCR structural biology arrived in 2011 when Brian K. Kobilka reported the first crystal structure of the agonist-induced β-2 adrenergic receptor (β2AR)-G_s_ protein signaling complex at near-atom resolution, which unveiled GPCR-mediated transmembrane signal transduction [11]. This breaking work was recognized internationally when Kobilka, along with Robert J. Lefkowitz, was awarded the 2012 Nobel Prize in Chemistry. However, despite these advances, obtaining well-diffracting crystals of GPCRs with ligands is time-consuming and challenging. Additionally, the majority of GPCR crystal structures solved thus far represent either inactive states or conformations that mimic active states rather than truly active ones. On a promising note, advancements in ultrafast time-resolved crystallography have paved the way for deeper insights into GPCR dynamics. A notable example is the utilization of this technology to elucidate the cascade of events through which photoactivated retinal triggers the activation process of rhodopsin, as referenced in [12].

The rise of cryo-EM technique has been a boon for GPCR research, especially for GPCR-signaling complexes, including the complexes of receptors with G-protein, arrestins, and other signaling mediators. It complements traditional techniques and offers new avenues to probe the relationship between GPCR structures and functions. This has been crucial in understanding the full spectrum of GPCR signaling pathways and paving the way for novel therapeutics. Cryo-EM has brought significant advancements to the structural biology of active GPCRs. To date, 151 receptors, which account for 60% of the total GPCR complex structures, have been resolved by the single-particle cryo-EM technique. Nevertheless, solving the cryo-EM structure of inactive GPCRs with antagonists presents inherent challenges. The primary hurdle stems from the relatively low molecular weight of such complexes, which can significantly compromise the signal-to-noise ratio in image processing. As a result, while cryo-EM offers transformative potential, its application for inactive GPCRs, especially those bound solely to antagonists, requires specialized approaches.

Recent strides in protein engineering have made much progress in determining the structures of inactive GPCRs using the cryo-EM method. These advancements can be broadly categorized into two strategic approaches. The first strategy employs the fusion of a rigid protein to the ICL3 region. Notably, Zhang et al. deciphered the structure of Smoothened receptor (SMO) at a remarkable global resolution of 3.7 Å by substituting ICL3 with the fusion protein *Pyrococcus abysii* glycogen synthase (PGS) (Fig. 2c) [13]. While the extended helix linking SMO and PGS typically lacks structural rigidity, the hydrophobic interactions between the two surprisingly augment the overall structural integrity. In addition, Gabriella Collu’s team introduced a rigid fusion protein, *AmpC* β-lactamase, to enhance both the molecular weight and stability of the β-1 adrenergic receptor (β1AR) [14].

The alternate strategy is the use of antibodies (nanobody or Fab) that can stabilize receptors. In a pioneering protein engineering study, the cryo-EM structure of the Frizzled-5 receptor (FZD5) was determined using antibodies against apocytochrome b562 RIL (BRIL) and the Fab nanobody (Fig. 2c) [15]. Following this, a research group independently resolved the inactive structure of GPR183 employing a similar approach [16]. Here, the rigidity between the BRIL fusion protein and the receptor, which forms extended helices for TM5 and TM6, proves critical. In a recent study, Guo’s team amalgamated previously mentioned strategies, employing a refined multipoint fusion approach (Fig. 2c) [17]. In the process of protein reconstruction, the BRIL fusion protein was inserted to substitute ICL3, and the ALFA helix tag was fused to the terminus of helix 8 (H8) [17]. A bivalent ‘glue’ molecule containing the anti-BRIL and anti-ALFA nanobodies was added to conjugate BRIL and ALFA and stabilize the cytoplasmic domain of the receptor [17]. To further enhance the rigidity of the intercellular region of the receptor, the E3/K3 coiled coil was introduced into this system [17]. In addition, the Nb6 nanobody was specifically designed to target the ICL3 region of the κ-opioid receptor (κOR) (Fig. 2c) [18]. The ICL3 region replacement at other GPCRs could accelerate GPCR determination.

## The structural characteristics of GPCRs for ligand recognition and receptor activation

### Canonical and noncanonical activation mechanisms of class A GPCRs

**Fig. 3:**
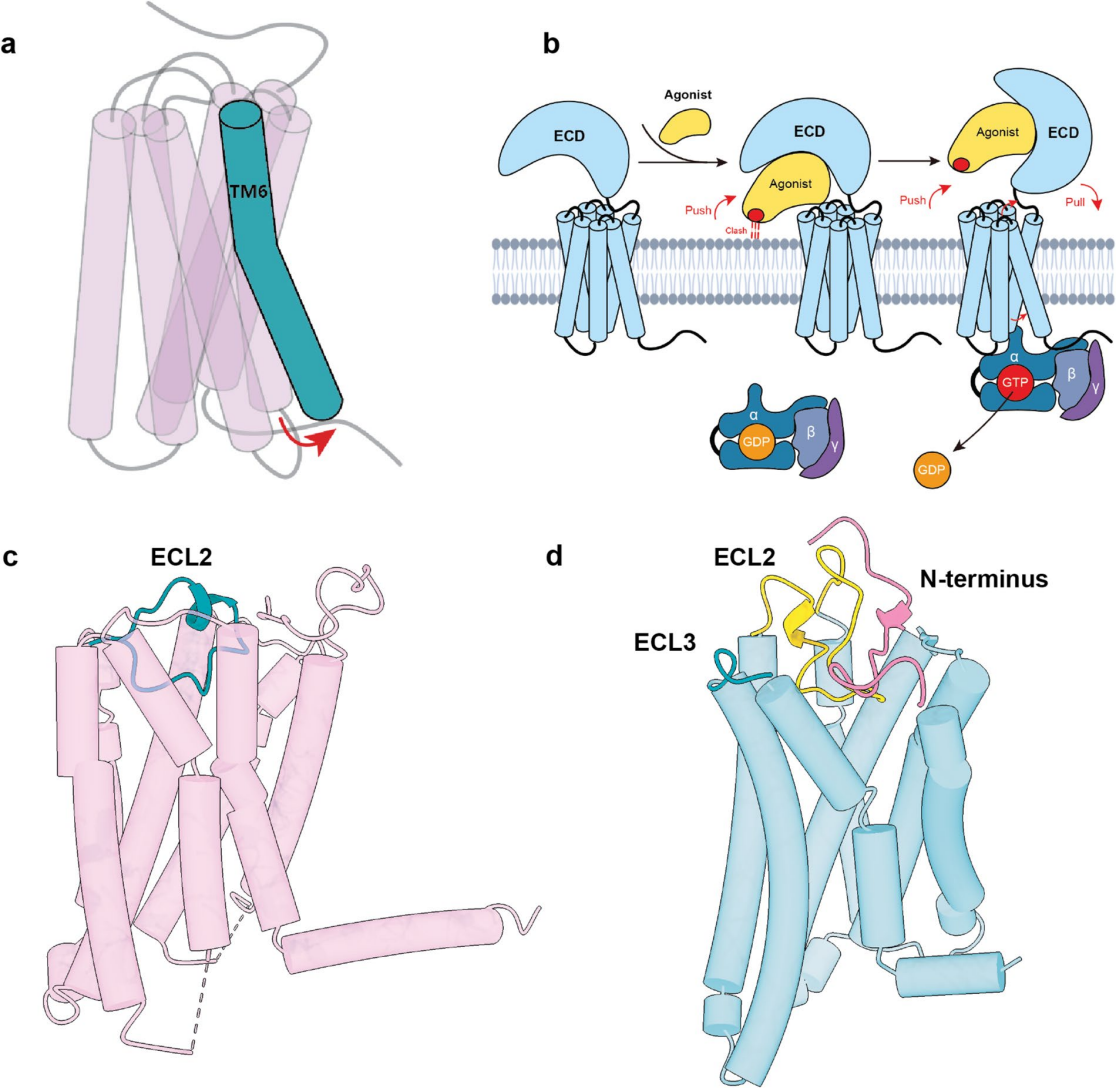
The structural features of Class A GPCRs. **a** Hallmark for Class A GPCR activation. The cytoplasmic region of TM6 moves outward during receptor activation. **b** The “push-pull” activation model of the glycoprotein hormone receptor subfamily. **c** The ECL2 region acts as a “built-in” agonist for GPR52 (PDB: 6LI2). **d** The N-terminus, ECL2 and ECL3 contribute to the activation of OR51E2 (PDB: 8F76)

Class A GPCRs, also known as the “rhodopsin-like family”, encompass various subgroups based on their ligand specificity. These subgroups include aminergic, peptide, protein, lipid, melatonin, nucleotide, steroid, alicarboxylic acid, sensory, and olfactory [5]. The pocket formed by the 7TM bundles serves as a binding site for orthosteric ligands, while the intracellular region is responsible for coupling with downstream effector proteins such as G-proteins and arrestins. Additionally, a conserved disulfide bond between ECL2 and TM3 contributes to the structural stability of the GPCR [19]. Upon activation, a serial conserved “micro switch” motif, including CWxP, Na^+^ pocket, PIF, DRY and NPxxY motif, is observed to exhibit conformation arrangement in GPCR [20–22]. When sensing the agonist, the collapse of the Na^+^ pocket (D^2.50^, S^3.39^, N^7.45^ and N^7.49^) in the 7TM core domain (7TMD) occludes the sodium ion, triggering the movement of TM7 toward TM3. In the intracellular region, residue Y^7.53^ in the NPxxY motif loses contacts with residues in TM1 or H8 and forms new contacts with residues in TM3, strengthening the packing of TM3 and TM7. In addition, the interhelical salt bridge between R^3.50^ and D^3.49^ in the DRY motif was disrupted. Collectively, the conformational arrangements of these conserved motifs result in notable outward displacement of TM5 and TM6 with respect to intracellular G-protein coupling (Fig. 3a).

In addition, certain class A GPCRs exhibit unique characteristics related to ligand binding and activation. A notable example is the glycoprotein hormone receptor subfamily (encompassing FSHR, LHR, and TSHR) of GPCRs [23–26]. These receptors contain a large extracellular domain (ECD) that primarily recognizes endogenous ligands, particularly glycoprotein hormones. When these hormones bind to the receptor, they trigger an upward rotation of the ECD, displaying a “push-and-pull” activation mechanism powered by glycoproteins (Fig. 3b). This dynamic alteration coincides with a significant conformational shift at the interface between the ECD and the 7TM bundles. Within this interface, particular attention is given to the p10 peptide, which acts as an intrinsic agonist. The shifting configuration of the p10 region crafts a space that allows the extracellular region of TM7 to move inward. This causes a distinct kink in the helix at positions M^6.48^ and D^6.44^. Notably, these residues are conserved across the glycoprotein hormone receptors and mirror the toggle switch residues W^6.48^ and F^6.44^ in classic GPCRs.

Most class A GPCRs are orphan receptors, since their endogenous ligands have not yet been discovered. Orphan GPCRs play important roles in physiological functions and are associated with various human diseases, such as schizophrenia, type 2 diabetes, attention deficit and hyperactivity disorder (ADHD), cognitive impairments, brain malformations, and Alzheimer’s disease. Approximately 75% of these receptors exhibit constitutive activity, and structure determination could help us to understand the mechanism of activation [27]. To date, approximately a dozen structures are available for orphan receptors. For example, the structures of GPR52, GPR21 and GPR17 reveal that the ECL2 region acts as a built-in agonist for receptor activation (Fig. 3c) [28–30]. Compared with G-protein bound A2A or β2 receptors, the cytoplasmic end of TM6 is much shorter in GPR21, which might suggest divergent G-protein coupling for orphan GPCRs [29]. Orphan GPCR research is still in its infancy, but as the number of reported receptor structures and in-depth studies, various mechanisms of constitutive activity and active conformation will continue to be revealed, new opportunities are emerging in this field.

Olfactory receptors sense odorants and can be classified into three subgroups, among which the odorant receptors (ORs) and trace amine-associated receptor (TAAR) families belong to Class A GPCRs. In addition to being discovered in olfactory sensory neurons (OSNs), they are also expressed in extranasal tissues and are involved in diverse biological processes, revealing potential therapeutic and diagnostic targets. Recently, two distinct research groups independently made structural breakthroughs in olfactory receptors that provide insight into odorant recognition and receptor activation. Aashish Manglik’s team reported the structure of Olfactory receptor 51E2 (O51E2R), a member of ORs, as well as mTAAR9 resolved by Sun’s team. The overall structures of these two receptors display similar architecture and activation hallmarks with the canonical Class A GPCRs [31]. However, a unique structural feature in which the N-terminus and ECL2 of the receptor form a conserved disulfide bond has been found in OR51E2 and mTAAR9 (Fig. 3d) [32]. This particular disulfide bond may contribute to odorant binding and play a critical role in receptor activation. In addition to structural features, the activation mechanism of OR51E2 is significantly distinct from that of canonical Class A receptors. Serial conserved “micro switch” motifs, such as CWxP and PIF, are absent in ORs. In the OR51E2 structure, the conserved FYGx^6.50^ motif in TM6 substituted the canonical CWxP motif, forming an extended hydrogen-bonding network between the residues of Y^6.48^, S^3.40^, R^4.52^ and D^5.50^ that leads to the outward movement of TM6 in the cytoplasmic end, similar to the arrangement of the canonical PIF motif. Furthermore, the rotation of R^6.59^, which is absent in Class II ORs adjacent to ECL3, triggers the activation of OR51E2 (Fig. 3d). These two studies elucidate the molecular mechanisms underlying the recognition and activation of two different groups of olfactory receptors, providing a theoretical and structural basis for more in-depth study of olfactory receptors and targeted drug development.

### Ligand recognition and receptor activation of class B GPCRs

Class B GPCRs are categorized into two subfamilies: B1 secretin receptors and B2 adhesion receptors. The B1 secretin family comprises 15 members. Their endogenous ligands are peptide hormones. B1 GPCRs play a pivotal role across a spectrum of physiological processes, such as blood sugar regulation (glucagon receptor-like family) [33–37], calcium modulation (parathyroid hormone & calcitonin receptors) [38–41], adrenal hormone control (corticotropin-releasing factor receptors) [42, 43], and gastrointestinal motility and secretion (vasoactive intestinal polypeptide (VIP) & pituitary adenylate cyclase-activating peptide (PACAP) receptors) [44–46].

**Fig. 4:**
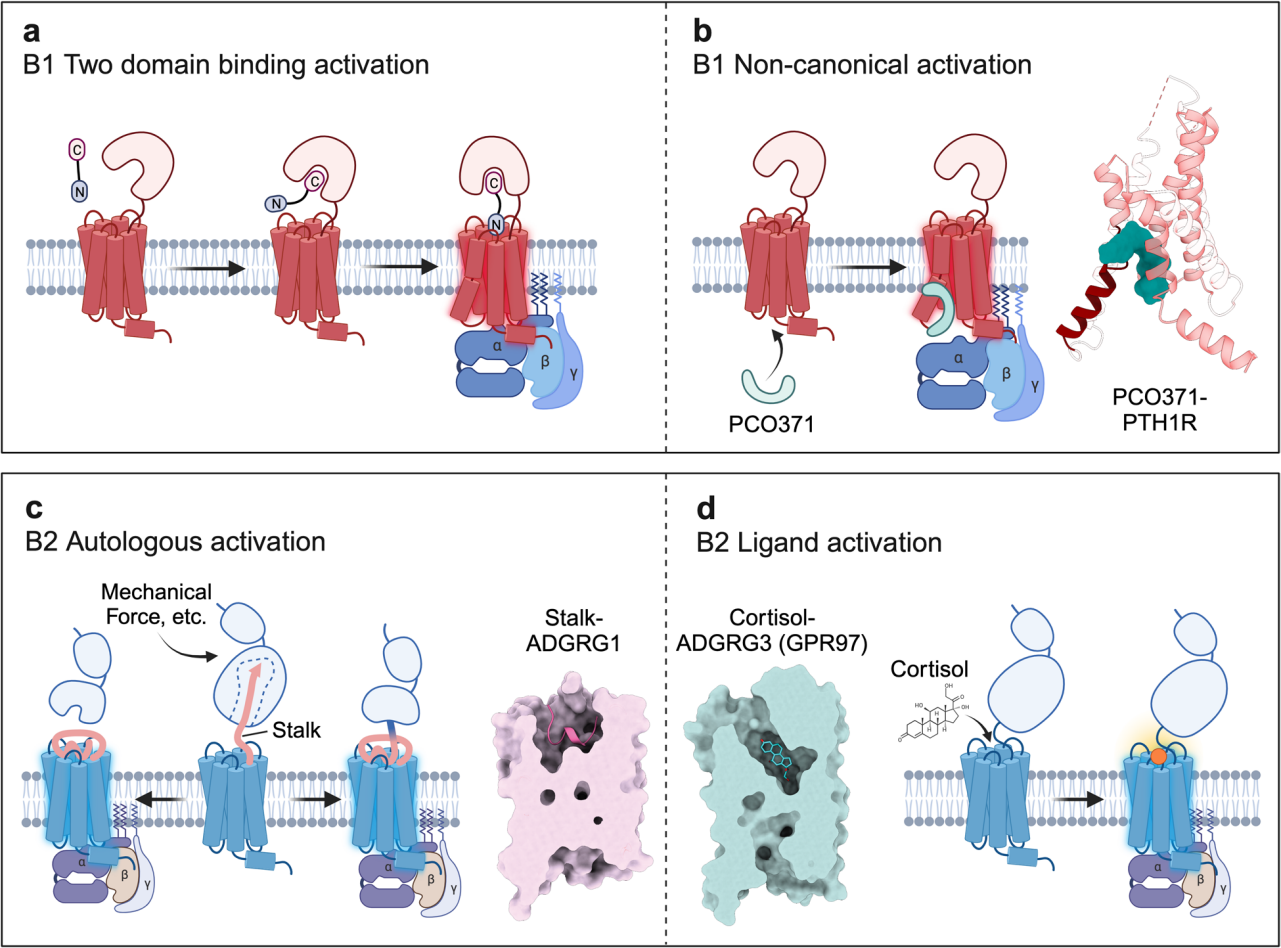
Activation mechanisms of Class B GPCRs. **a** Scheme of the “two domain binding model” as the common activation mechanism of Class B1 receptors. **b **PCO371 binds to the intracellular pocket (e.g., PCO371-PTH1R-G_s_ complex, PDB: 8GW8) of Class B1 receptors and reveals a noncanonical activation mode. **c** Stalk undergoes a transition from a β-sheet to a partial α helix when mediating autologous activation of aGPCRs. Binding of the α helical stalk in the ligand pocket of ADGRG1 is from the stalk-ADGRG1-miniG13 complex (PDB: 7SF8). **d** Cortisol binds to the orthosteric pocket of ADGRG3 (GPR97) and triggers receptor activation as the endogenous ligand. The binding of cortisol in the ligand pocket of ADGRG3 is from the cotisol-ADGRG3-miniG_o_ complex (PDB: 7D77)

B1 receptors share a common architecture consisting of an extracellular domain (ECD) with 120-160 residues and a transmembrane domain (TMD) formed by seven helices. Both domains collaboratively engage in ligand recognition, which is acknowledged as the ‘two-domain binding model’ of the B1 GPCR activation scheme (Fig. 4a) [33, 47]. The ECD of the receptors rapidly recognizes and binds the C-terminus of the ligand, establishing the initial ligand‒receptor specificity. Subsequently, the N-terminus of the ligand penetrates the orthosteric pocket. This interaction induces receptor conformational changes, facilitating the recruitment of downstream G-proteins, and represents the rate-determining step in receptor activation [48].

Class B1 GPCRs undergo a series of conserved conformational changes within the 7TMD. These changes are characterized by an outward movement of the extracellular side of TM6, TM7, and ECL3, coupled with an inward movement of the extracellular side of TM1, forming a V-shaped pocket conducive for ligand binding [49]. The common motif P^6.47b^-××-G^6.50b^ (superscripts refer to the Wootten numbering system for class B GPCRs) [50] induces a kink in the middle of TM6 upon activation. This facilitates the outward movement of the intracellular end of TM6, thereby opening the intracellular pocket for G-protein coupling. The B1 receptor family couples to a variety of G-protein subtypes to activate downstream signaling, with G_s_ being the predominant subtype [51].

A recent study identified a conserved pocket within the intracellular regions of TM2/3/6/7 of Class B1 receptors [52, 53]. The small molecule PCO371 binds to this site, prompting the intracellular side of TM6 to swing outward, thus stabilizing the receptor in an activated state conducive to G-protein coupling even in the absence of orthosteric ligands (Fig. 4b). Notably, 7 out of 15 B1 receptors can be activated by PCO371, indicating the diverse activation mechanisms inherent to the B1 family.

The B2 adhesion G protein-coupled receptor (aGPCR) family comprises 33 members. These receptors participate in fundamental physiological processes such as tissue development, reproduction, cerebrocardiovascular function, and endocrine regulation. Mutations or aberrant expression of these receptors are directly linked to diseases, including reproductive and neurodevelopmental disorders, as well as tumors [54, 55]. For instance, ADGRB3 plays a crucial role in synapses within the hippocampus and cerebellum, and its dysfunction has been proven to be associated with schizophrenia [56]. ADGRD1 (GPR133), ADGRG1 (GPR56), and ADGRG5 (GPR114) sense mechanical forces through their N-terminal extracellular domain, thereby participating in cell-cell contact and maintaining cellular homeostasis [57–59]. ADGRE1 and ADGRE5 are prominently expressed on immune cells and are involved in various immune responses, such as neutrophil migration and phagocytosis [60, 61]. Additionally, associations have been drawn between ADGRF1 (GPR110) and breast cancer progression [62], as well as ADGRL3 and attention-deficit/hyperactivity disorder [63, 64]. Mutations in ADGRV1 have been implicated in Usher syndrome type 2C, leading to deafness and blindness [65]. Thus, understanding the structural characteristics and operational modes of aGPCRs holds promise for revealing the molecular basis of biological processes, disease mechanisms, and the development of novel therapeutic strategies.

aGPCRs exhibit distinctive structural features, encompassing a multidomain N-terminal extracellular region and the GPCR autoproteolysis-inducing (GAIN) domain, which contains the conserved GPCR proteolysis site (GPS) [54, 66].

Predominantly, aGPCRs hydrolyze at the GPS site spontaneously, yielding two distinct fragments: the extracellular N-terminal fragment (NTF) and the C-terminal fragment (CTF), which contains seven transmembrane helices. These fragments, NTF and CTF, are observed to maintain a noncovalent association on the cellular surface post hydrolysis [67–69].

The sequence at the N-terminus of CTF can act as an agonist to activate the receptor and recruit downstream G-proteins. This sequence is referred to as the “Stalk” (also known as “Stachel” or “tethered agonist”) [70–74]. aGPCRs exhibit an inherent capacity for self-activation through Stalk, displaying high constitutive activity. Upon activation, the Stalk sequence undergoes a conformational shift, transitioning from a β-sheet to a partial α-helical loop, which then engages with the orthosteric pocket (Fig. 4c). The Stalk predominantly interacts with the TMD via its hydrophobic residues. Notably, it is not imperative for proteolysis to form a free Stalk sequence. Even while tethered to the GAIN domain, it possesses the capability to activate the receptor in a similar binding fashion [70, 73].

Beyond the intrinsic Stalk-mediated activation triggered by mechanical force, the ADGRG subfamily within aGPCRs demonstrates a capability for steroid recognition, including glucocorticoids, progesterone, and testosterone (Fig. 4c) [75, 76]. This suggests that aGPCRs, in addition to their self-activation mode, are also endowed with endogenous ligands that can engage directly with the 7TM core.

Despite the low sequence similarity among the aGPCR family, there remains a consistent theme in the conformational changes of the transmembrane helices upon activation. The entry of Stalk into the orthosteric pocket induces an outward deflection of the extracellular facets of TM6 and TM7. G^6.50b^ and G^7.50b^ are central amino acids contributing to this conformational change, with W^6.53b^ serving as the activation toggle switch [70–73]. In contrast to autologous activation, the agonism of steroids, such as cortisol, elicits a subtler outward shift of the extracellular aspects of TM6 and TM7, anchoring the receptor in an intermediate active state (Fig. 4d). This is distinct from the Stalk sequence-mediated self-activation, which represents a fully activated state of the receptors, reflecting that the Stalk sequence acts as a full agonist [71, 77].

### Architectural features of inactive and active states of class C GPCRs

Class C GPCRs are subdivided into four groups based on their specific agonists: calcium-sensing receptor (CaSR), amino acid receptors (including γ-aminobutyric acid receptors GABA_B1_/GABA_B2_ and metabotropic glutamate receptors (mGluRs), taste receptors (Taste 1 receptors 1-3), and orphan receptors [78]. The advent of cryo-EM technology has revolutionized our understanding of Class C GPCRs. By offering near-molecular-level resolution, it lays the groundwork for comprehending their mechanisms of activation and functionality. The structural characteristics and signal transduction of CaSR, GABA_B_, and mGluRs have been more extensively studied, yet there remains much to uncover about Class C GPCR intricate structures and receptor dynamics.

**Fig. 5:**
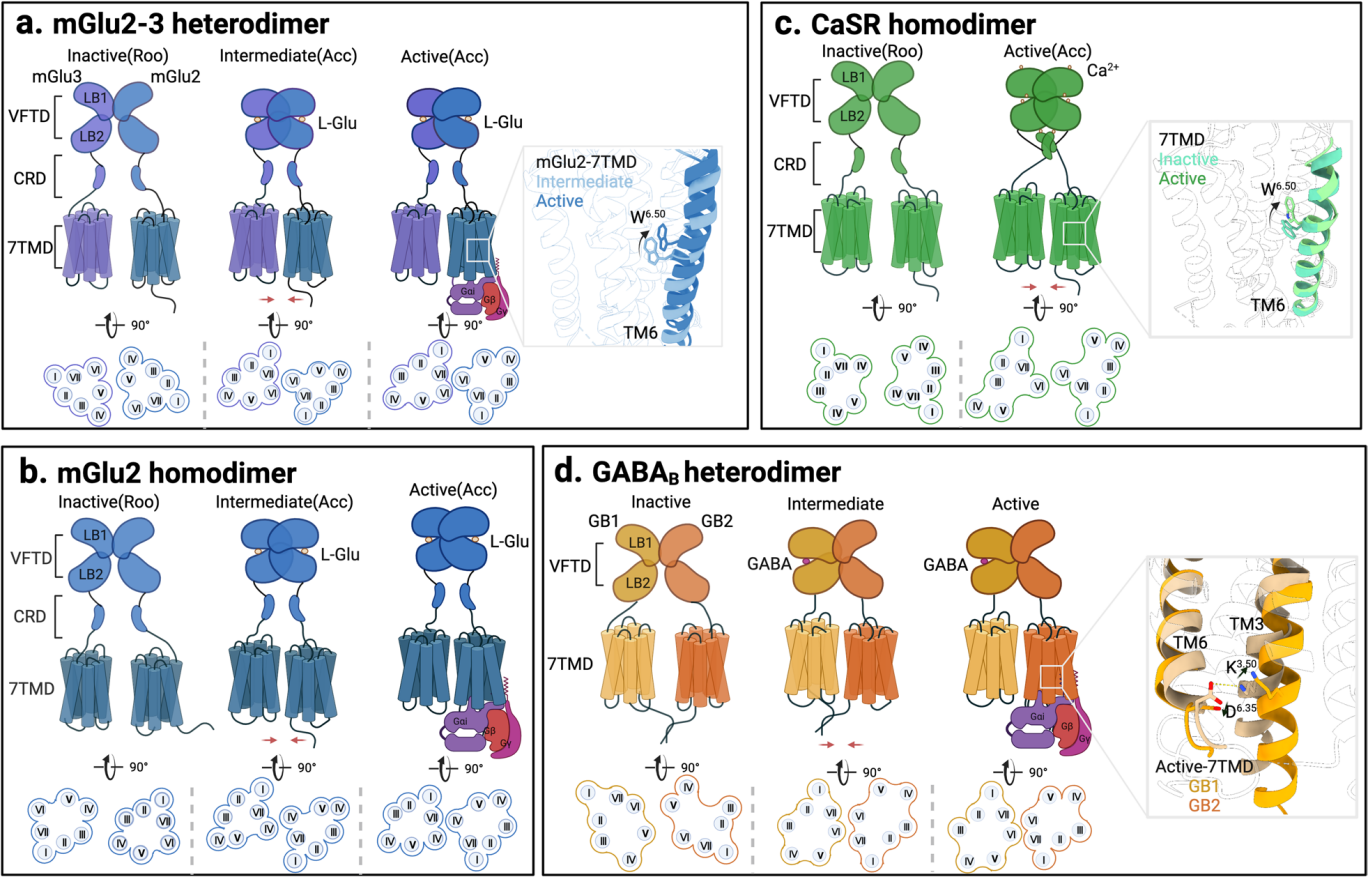
General activation mechanisms of Class C GPCRs. Class C GPCRs are color coded as follows. The mGlu2, mGlu3, CaSR, GB1 and GB2 subunits are blue, light purple, green, yellow and orange, respectively. The endogenous agonists L-Glu, Ca^2+^, and GABA are indicated by colored balls; the G protein heterotrimers Gαi, Gβ, and Gγ subunits are indicated by purple, red, and fuchsia, respectively. The general conformational alterations of the class C GPCR dimer are shown above, the intracellular view of the TMD is displayed below, and the conformation of key amino acids is presented on the right. **a** Structures of the mGlu2-3 heterodimer in the inactivated state (PDB: 8JCV), intermediate activated state (PDB: 8JD2), and fully activated state (PDB: 8JD3). **b** Structures of the mGlu2 homodimer in the inactivated state (PDB: 7EPA), intermediate activated state (PDB: 7EPB), and fully activated state (PDB: 7E9G). **c** CaSR homodimer in the inactivated state (PDB: 7M3J) and the structure of the activated state (PDB: 7M3G). **d** Structures of the GABA_B_ heterodimer in the inactivated state (PDB: 6VJM), the intermediate activated state (PDB: 6UO9), and the fully activated state (PDB: 7EB2)

Class C GPCRs are distinguished from other GPCRs by two structural features: large ECDs and constitutive dimerization (Fig. 5a and d) [78]. CaSR, GABA_B_ and mGluRs are characterized by an extracellular domain (ECD) comprised of a large bilobed “clamshell” domain [78]. Their ECD contains a venus fly trap domain (VFTD) for ligand binding, which is further subdivided into upper lobe (LB1) and lower lobe (LB2) domains [78]. For GPR158, the ECD contains a cache domain (a name derived from “calcium channels and chemotaxis receptors”) for ligand binding [79]. For both CaSR and mGluRs, their ECD additionally incorporates a cysteine-rich domain (CRD) that bridges the VFTD and the 7TMD, while GABA_B_ is structured with a VFTD directly connected to the 7TMD by a more rigid linker (Fig. 5a and d) [78].

CaSR, GABA_B_, and mGluR dimers generally share a common activation mechanism [80–84]. In their resting state, the VFTD of these receptors adopts an open conformation. VFTD dimerization is orchestrated by the LB1-LB1 interaction [85–87], while the 7TMD-level dimerization interface is versatile, e.g., the TM5/6-TM5/6 interface for CaSR [80], TM3/5-TM3/5 for GABA_B_ [88], and TM5-TM5 interface for mGluRs [89]. Upon agonist binding to the VFTD cleft, it engages with residues within both LB1 and LB2, prompting closure of the VFTD. This structural shift enhances the LB2-LB2-mediated dimerization interface for CaSR and GABA_B_ [85, 86] or shortens the LB2-LB2 distance for mGluRs [87], inducing rotation and convergence of the two subunits, further leading to the formation of the TM6-TM6 interface (Fig. 5a and d) [80, 82, 89]. This process activates the 7TMD, allowing one of the subunits to couple with the G protein heterotrimer, culminating in the full activation of the receptor.

CaSR stands alone as a unique member of its subfamily. Functionally, it operates as a homodimer and primarily couples with G_q_. There are four binding pockets of the agonist Ca^2+^ on each subunit of the CaSR dimer. Beyond the dual binding sites nested within the VFTD cleft, Ca^2+^ shows affinity for the apical loop region of LB1 and establishes an association at the LB2-LB2 dimer interface. This binding paradigm bridges the LB2 of one subunit with the CRD of its counterpart, thereby inducing a conformational change in the CRD [90]. The CRD further relays activation signals to the 7TMD via its interplay with ECL2. The key residue of CaSR activation, W818^6.50^, undergoes a dramatic conformational shift, pivoting its side chain from an external to an internal orientation and gravitating toward the 7TM core (Fig. 5c). Further activation of the 7TMD for G-protein coupling is asymmetric. At the 7TM interface, TM6 of 7TM^A^ sits higher than the opposing TM6 of 7TM^B^, which is tilted relative to 7TM^A^ (Fig. 5c). It is also reflected by the same 7TM positive allosteric modulators (PAMs), either “evocalcet” or “cinacalcet”, assuming distinct poses in the two protomers. In 7TM^A^, the PAMs adopt an extended conformation, whereas in 7TM^B^, they are bent. Experiments also corroborate that the specific conformation of asymmetric 7TMD dimerization favors G_q_ coupling [80].

GABA_B_ functions as a heterodimer with two subfamily members, GB1 and GB2. While GB1 is responsible for agonist binding, GB2 facilitates coupling with the G protein heterotrimer [81]. Notably, an ionic lock formed between K^3.50^ and D^6.35^ in GABA_B_ stabilizes its inactive state [82]. The heterodimer interface between the TM5 and TM3 helices of both subunits embodies the signature of the inactive conformation of the GABA_B_ receptor. This interaction from the TM3 and TM5 helices (H572^3.55^ and E673^5.60^ in GABA_B1_; H579^3.55^ and E677^5.60^ in GABA_B2_), defined as the “intersubunit latch”, preserves the transmembrane orientation of the dual subunits in the inactive state [88]. During activation, the agonist GABA exclusively binds to the VFTD cleft of GB1, triggering the closure of the GB1-VFTD, whereas the GB2-VFTD remains in an open conformation (Fig. 5d) [86]. However, the closure of GB1-VFTD is sufficient to induce a conformational change in GB1-7TMD and GB2, enhancing the LB2-LB2 interaction and establishing the TM6-TM6 dimerization interface (Fig. 5d) [82]. Upon activation of the 7TMD, the ionic lock between K^3.50^ and D^6.35^ in GB1 persists, whereas in GB2, this ionic lock is disrupted due to the increased distance between the intracellular ends of TM3 and TM5 upon receptor activation (Fig. 5d) [82]. Nevertheless, the movement of TM3 and TM5 creates ample space to accommodate the G-protein, enabling GB2 to couple with the G_i_ heterotrimer and activate downstream signal transduction [81, 82].

The mGluR family encompasses eight members, mGluR1-8, and is categorized into three distinct groups. Group I consists of mGluR1 and mGluR5, which mainly couple with G_q_ protein heterotrimers. Group II comprises mGluR2 and mGluR3, while Group III includes mGluR4, 6, 7, and mGluR8, both of which primarily couple with G_i_ heterotrimers [91]. Although mGluRs predominantly function as homodimers, functional heterodimers such as mGluR1-5, mGluR2-3, mGluR2-4, and mGluR2-7 have also been identified [92, 93]. When mGluRs bound to an agonist without G-protein coupling, their 7TMD displays a symmetric activated conformation mediated by the TM6-TM6 dimer interface (Fig. 5a and b) [84, 89, 94, 95]. W^6.50^ acts as the activation switch for mGluRs. Following coupling with G-protein heterotrimers, the 7TMD exhibits a fully activated asymmetric conformation mediated by the TM1/5/6/7-TM6 dimer interface (Fig. 5a and b) [83]. In this scenario, the subunit contributing TM1/5/6/7 to the dimer interface remains uncoupled from the G-protein heterotrimer, whereas the subunit contributing TM6 is coupled to the G_i_ protein heterotrimer (Fig. 5a and b) [83]. In the context of the heterodimer, exemplified by mGlu2-4 pairing, the agonist-bound state without G-protein coupling reveals an asymmetric dimer interface at the 7TMD, orchestrated through TM1/5/6/7-TM6 interactions [95]. This presents a distinct contrast to the mGluR homodimers devoid of G-protein coupling. However, it bears resemblance to the dimerization interface in G_i_-coupled mGlu2 or mGlu4 homodimers, underscoring the absence of a stable symmetric dimerization interface during the activation of heterodimers [95]. The diverse dimeric forms of mGluRs contribute to their intricate functional and pharmacological properties, warranting further exploration in future studies.

### Conformation arrangements in Class F GPCR activation

Class F GPCRs comprise one SMO and Frizzled family receptors (FZDs) [96]. SMO is primarily involved in the Hedgehog (Hh) signaling pathway and is essential in homeostasis maintenance and tissue repair [97]. The FZD family consists of 10 receptors, which can be further divided into 5 subfamilies based on sequence homology and their recognition specificity for the endogenous ligand Wnt: FZD1/2/7, FZD3/6, FZD4, FZD5/8, and FZD9/10 [98]. FZDs play a crucial role in embryonic development, stem cell regulation, and tissue homeostasis [99, 100], while their dysfunction has been implicated in various tumors, including colon [101], breast [102, 103], and ovarian cancers [104], highlighting their potential as therapeutic targets.

**Fig. 6:**
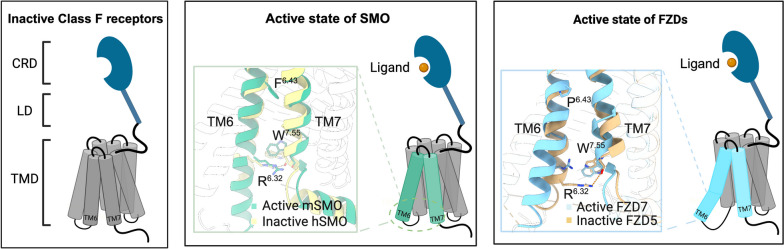
Conformational alterations during Class F receptor activation. Class F receptors are composed of a cystine-rich domain (CRD), linker domain (LD) and transmembrane domain (TMD), and the inactive state is shown on the left. When activated by the ligand, TM6 in SMO and FZDs undergoes an outward shift, and a hydrogen bond between the conserved residues R^6.32^ and W^7.55^ in the inactive receptor is disrupted. The difference between SMO and FZDs is that the TM6 in SMO exhibits a parallel outward shift, while FZDs achieve a similar displacement of its cytoplasmic segment through a helical kink. This difference may be caused by the conserved residue P^6.43^ in the FZDs (as opposed to F^6.43^ in SMO). PDB ID of these structures: active mSMO (6O3C), inactive hSMO (5I7D); active FZD7 (7EVW), inactive FZD5 (6WW2)

Compared to other GPCRs, Class F GPCRs have a cysteine-rich domain (CRD) at the N-terminus, which is connected to the conserved 7TM domain via a linker (Fig. 6) [96]. Notably, the CRD serves as the recognition region for endogenous ligands (cholesterol for SMO and Wnt for FZDs) and is involved in receptor activation and downstream signaling initiation [96], emerging as a hotspot for drug development [105]. The CRD contains three ligand binding sites. While the lipid-binding groove (site 1) is present in all Class F GPCRs, FZD CRDs also possess two additional ligand-binding sites (sites 2 and 3) that are absent in SMO [96].

Several active structures of SMO have been elucidated to date [106–109]. Insights from the complex of G_i_ heterotrimer-coupled human SMO (hSMO) (PDB: 6OT0) [106] and the agonist SAG21k-bound mouse SMO (mSMO) with a stabilizing nanobody NbSmo8 (PDB: 6O3C) [107] reveal a consistent receptor activation mechanism marked by analogous structural rearrangements and shared molecular switch dynamics.

In comparison to the inactive state, both structures reveal outward movement of the intracellular end of TM6 and inward movement of TM5 in the activated SMO, specifically a 7-8 Å outward shift for TM6 and a 4-5 Å inward shift for TM5 [108]. Additionally, in the active state of mSMO bound to the nanobody NbSmo8, the entire ECL3-TM6 helix shows a 3 Å displacement toward the extracellular side compared to its position in inactive mSMO [107]. The binding of cholesterol in the CRD induces an upward shift in ECL3 toward the CRD and directly relays to TM6 [107]. This provides insight into the role of the CRD in regulating SMO activation. Notably, the activated SMO structure, with its intracellular outward movement of TM6, mirrors similar structural shifts observed in Class A and B GPCRs [11, 40, 110].

The conserved residues R^6.32^ and W^7.55^ (superscript numbers refer to the Ballesteros and Weinstein numbering system) in Class F GPCRs were previously considered molecular switches for receptor activation [111], characterized by a hydrogen bond observed between R^6.32^ and W^7.55^ in the inactive conformation (Fig. 6). In contrast to the inactive state, the activation of SMO is accompanied by the disruption of polar interactions between R^6.32^ and W^7.55^, leading to a conformational rearrangement of TM6 (Fig. 6) [106, 107]. A similar mechanism is also evident in the FZD7-G_s_-Nb35 complex [112].

Wnt serves as an endogenous ligand for FZD receptors, orchestrating downstream Wnt/β-catenin signaling pathways through the formation of ternary complexes with coreceptors LRP5/6 [98]. Comparative analyses of the *Xenopus* Wnt8 (xWnt8) and human Wnt3 (hWnt3)-bound mFZD8 CRD structures [113, 114] revealed that the receptor recognition pattern of hWnt3 is nearly identical to that of xWnt8. Wnt displays a unique dual-domain structure, resembling a “hand” with an outstretched “thumb” and “index finger”, to grasp the two distinct binding sites of FZD8-CRD. One site is dominated by the palmitoylation Ser187, extending from the Wnt “thumb” tip into the deep groove of FZD8-CRD. The second site is situated opposite site 1, where the conserved tip of Wnt’s “index finger” (residues Cys315-Cys325) forms hydrophobic contacts within a recessed region between interhelical loops on the CRD. Within site 2, the finger loop positions hydrophobic residues, including Cys315, Phe317, Trp319, a unique tandem Cys320-Cys321 disulfide bond, and Val323, to establish primary van der Waals interactions with both mainchain and nonpolar residues of FZD8-CRD. The conservation of amino acids across both interfaces appears to facilitate a mechanism that underpins ligand-receptor cross-reactivity. Furthermore, the xWnt8-mFZD8 structure reveals that Wnt and Frizzled CRD can assemble into a 2:2 complex, which potentially has impacts on downstream signaling [114].

In 2021, the constitutively active structure of the FZD7-G_s_-Nb35 complex was elucidated [112]. When comparing the FZD7-G_s_ structure with the inactive FZD4 (PDB: 6BD4) [115] and FZD5 (PDB: 6WW2) [15], there was a notable outward curvature of TM6 and an inward displacement of TM5 on the cytoplasmic side. This conformational change mirrors that seen during SMO activation. The difference is that TM6 in the FZD7-mG_s_ complex achieves a similar displacement of its cytoplasmic segment through a helical kink [112], while TM6 in SMO-G_i_ exhibits a parallel outward shift compared to its inactive state (Fig. 6) [106, 107]. The MD simulations indicate that this difference may be caused by the conserved residue P^6.43^ in the 10 FZDs (as opposed to F^6.43^ in SMO) (Fig. 6) [111, 112]. In conclusion, SMO demonstrates a tendency for a straight TM6 in both ligand binding and functional readouts, whereas FZDs exhibit a kinked TM6 upon activation due to the presence of residue P^6.43^ [111]. These divergent activation mechanisms may provide insights beneficial for targeting Class F receptors to design drugs with better selectivity and pharmacological attributes.

### The activation mechanism of TAS2R46

Gustation is a sensory system used to prevent the intake of harmful substances and consists of five tastes: sour, sweet, bitter, salty, and umami, while the perception of bitter, sweet and umami is mediated by GPCRs [116–118]. Bitter taste recognition primarily involves TAS2Rs, which constitute a distinct class T GPCR subfamily characterized by their low sequence identity compared to other GPCRs [119–122].

TAS2Rs expressed in extraloral tissues represent potential drug targets for addressing conditions such as obesity, asthma, diabetes, and metabolic diseases [123, 124]. Previous research has revealed that TAS2Rs are expressed in enteroendocrine cells and play a pivotal role in appetite reduction. Specifically, TAS2Rs influence the release of orexigenic gut hormones and modulate intestinal movement upon detecting bitter compounds [125, 126]. Moreover, bitter agonists have been found to alleviate certain asthma symptoms by inhibiting the release of inflammatory factors in leukocytes and promoting relaxation of airway smooth muscles [127].

**Fig. 7:**
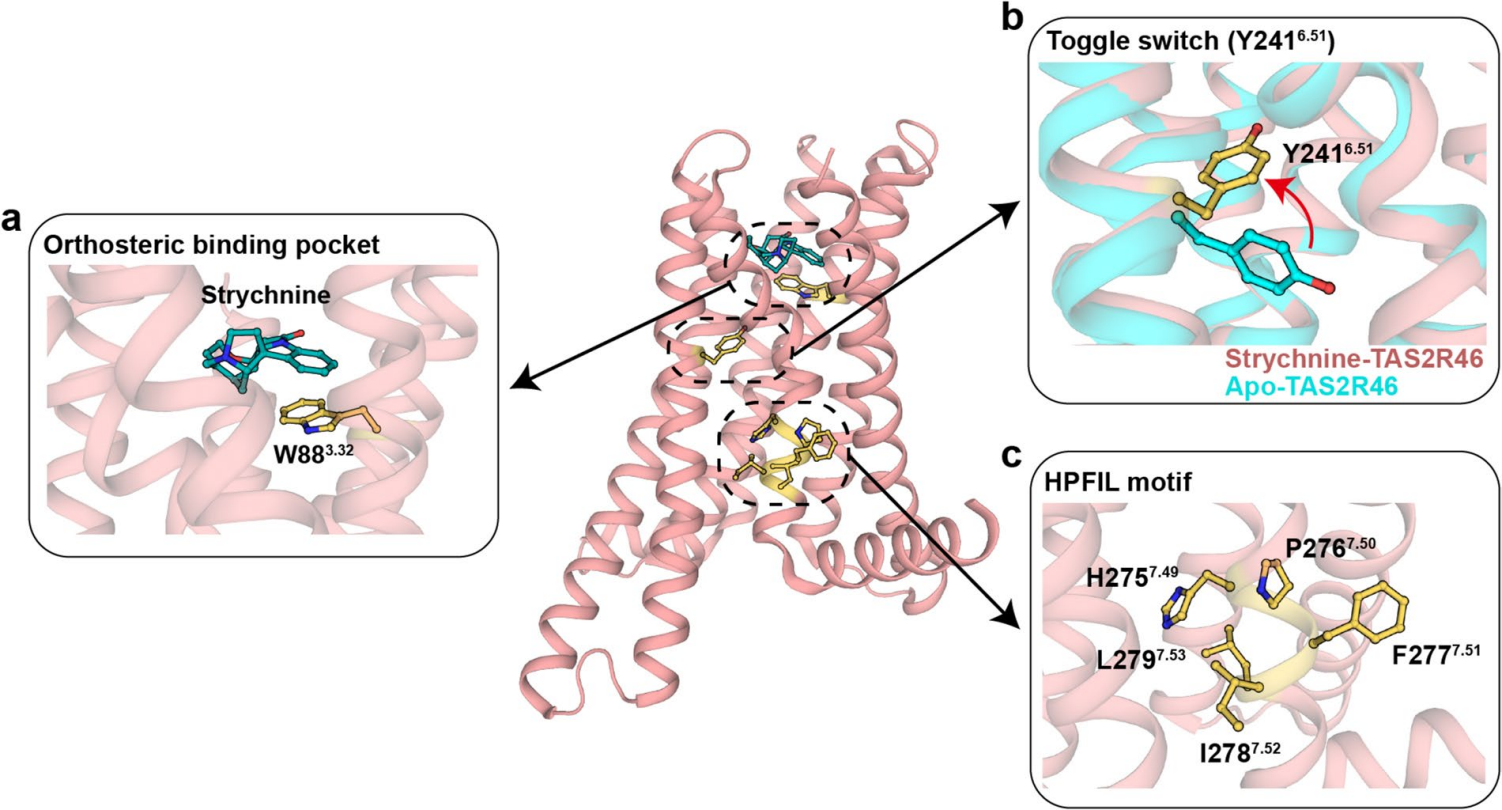
Structural features of TAS2R46. **a** Orthosteric binding pocket of TAS2R46 (PDB: 7XP6). The indole ring of W88^3.32^ is horizontally parallel to the benzene ring of strychnine. **b** The conformational changes of the “toggle switch” Y241^6.51^ between apo-TAS2R46 (PDB: 7XP4) and strychnine-TAS2R46; its side chain changed from pointing outward to pointing toward the core of the transmembrane helix. **c** The HPFIL motif in strychnine-TAS2R46

To date, only one TAS2R structure has been reported. Structural analysis of strychnine-bound TAS2R46 and its apo-form indicates that ECL2 adopts a short helical conformation, occupying the orthosteric binding pocket, similar to GPR52 (Fig. 7) [128]. However, there are notable distinctions in the activation mechanism of TAS2R46 compared to Class A GPCRs. Notably, in Class A GPCRs, the W^6.48^ residue within the CWxP motif serves as a “micro switch” during activation, whereas TAS2R46 features a cysteine at this position, which is not necessary for TAS2R46 activation [128]. Structural superimposition with C-X-C chemokine receptor type 2 (CXCR2), the most structurally similar to TAS2R46 in Class A GPCRs, reveals that the residue corresponding to W^6.48^ in TAS2R46 is Y241^6.51^ [128]. During TAS2R46 activation, Y241^6.51^ acts as a “toggle switch,“ undergoing an approximately 90° rotation, shifting from an outward orientation to pointing toward the core of the transmembrane helix (Fig. 7b) [128]. Although Y241^6.51^ plays a role akin to W^6.48^ in TAS2R46 activation, its rotation does not induce the outward movement of TM6, which distinguishes TAS2R46 from Class A GPCRs [128].

Furthermore, TAS2R46 lacks certain conserved motifs involved in the activation mechanism of Class A GPCRs, such as the NPxxY motif and the DRY motif. Instead, TAS2R46 features the HPFIL motif, and the residues within this motif participate in a hydrophobic interaction network that mediates the packing of TM3, TM6, and TM7 (Fig. 7c). This interaction mode differs from the role of the NPxxY motif in Class A GPCRs [128].

## GPCR signal transduction

GPCRs, as their name implies, couple G-proteins in the membrane when bound with agonists. The G-protein is composed of three subunits: Gα and Gβ, Gγ, forming a stable dimer. The activated GPCRs trigger the recruitment of inactive G-protein (the Gα subunit bound with GDP), leading to the exchange of GDP by GTP. Activated Gα dissociates from the Gβγ dimer and from GPCRs. G-proteins can be divided into four subgroups, G_s_, G_i_, G_11/q_ and G_12/13,_ according to the function of Gα [129, 130]. In contrast to the G-protein signaling pathways, GPCRs also trigger arrestin signaling and other noncanonical pathways. The different signaling pathways meditate distinct physiological or pathological processes.

### G-protein-mediated signaling pathway of GPCRs

Typically, Gα_s_ stimulates adenylyl cyclase (AC) and improves the second messenger cyclic adenosine monophosphate (cAMP) level in cells. In contrast, Gα_i_ inhibits adenylyl cyclase (AC) and decreases cAMP levels [131]. Gα_11/q_ activates phospholipase C (PLC), which catalyzes the conversion of phosphatidylinositol bisphosphate (PIP2) into diacylglycerol (DAG) and inositol 1,4,5-trisphosphate (IP3) [132]. The released DAG activates protein kinase C (PKC), while IP3 diffuses to the endoplasmic reticulum (ER) and binds to IP3 receptors on ligand-gated calcium channels on the surface of the ER, leading to a massive release of calcium ions into the cytosol [133]. The evaluated levels of Ca^2+^ activate Ca^2+^ and calmodulin-dependent protein kinase II (CaMKII) [134]. Gα_12/13_ stimulates Rho GTPase replacement by second messengers [135]. Meanwhile, the dissociation of the Gβγ dimer can also regulate numerous molecules (e.g., GIRK channels, TRPM3 and CaV) [136].

#### G-protein subtype selectivity of GPCRs

**Fig. 8:**
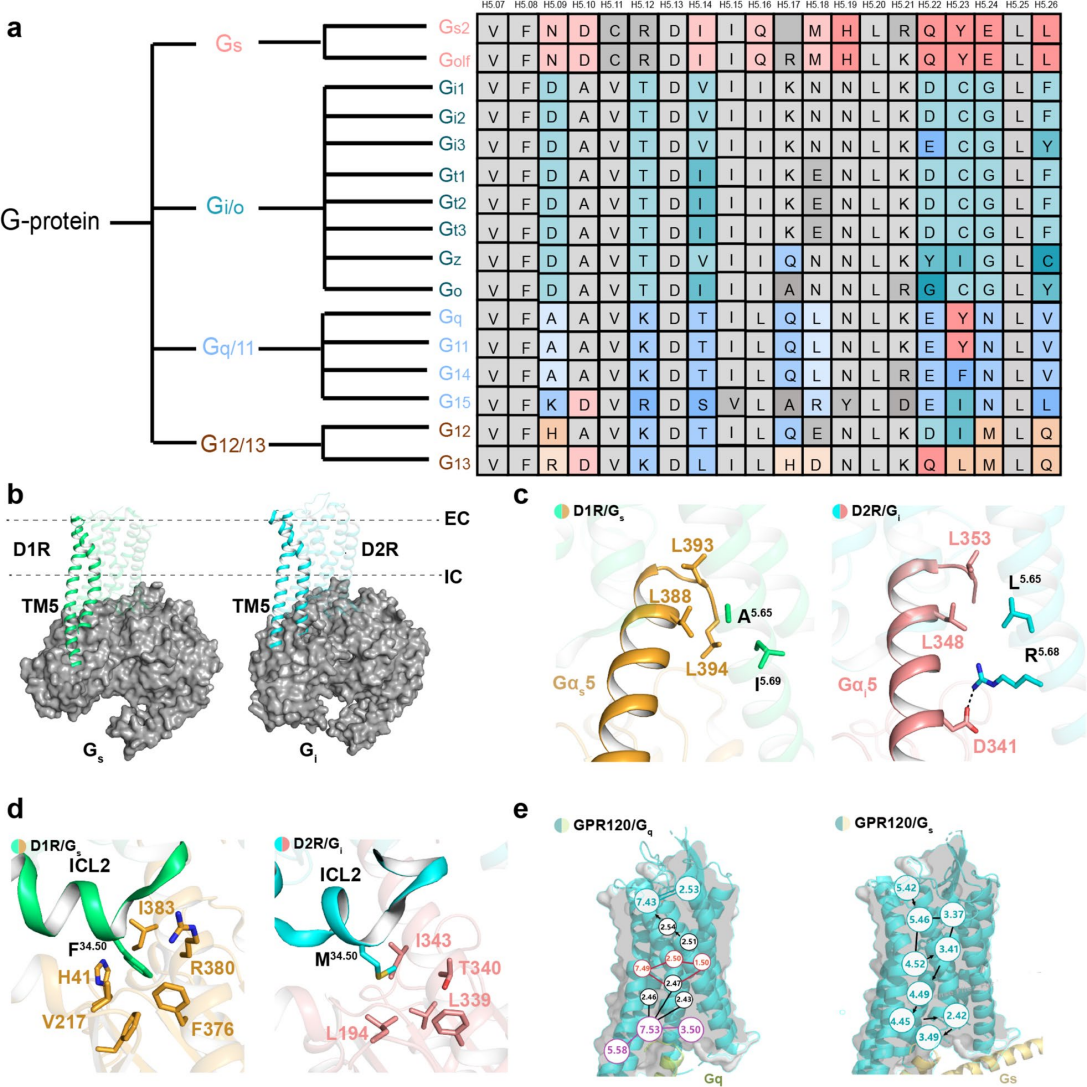
The elements in receptors determine the G-protein subtype selectivity. **a** Classification and sequence alignment of the C-terminus of the Gα subunit family. **b**, **c**, **d** Structural comparison of G_s_ (left)-D1R (PDB: 7CKW) and G_i_ (right)-D2R (PDB: 7JVR). **b** TM5 of D1R is longer than D2R in the cytoplasmic region. **c**. The distinct motifs in TM5 contribute to G-protein subtype selectivity between D1R and D2R. **d** Detailed interaction between the residue at the 34.51 position of ICL2 and Gα-proteins in G_s_ (left)- and G_i_ (right)-bound dopamine receptors. **e** Schematic diagrams displaying the propagating pathways that contribute to G-protein subtype-biased signal transduction for GPR120

The GPCR complex structures reveal that the regions TM3, TM5-7 and ICL2 are involved in G-protein heterotrimer coupling, especially for the C-terminal α5 helix in Gα. Sequence alignment of the Gα subunit suggests that sequence diversity is observed in the C-terminus. Previous studies reported that the C-terminus, also known as “the wavy hook”, plays a critical role in G-protein selectivity (Fig. 8a) [137–139]. In the α5 helix, the residue is Y in position H5.23 for G_s_ and G_q_ and smaller residues for G_i/o_ and G_12/13_, suggesting that the α5 helix also contributes to G-protein selectivity. Additionally, previous studies imply that the residues at position 34.51 of ICL2 play a critical role in G_i_ and G_s_ selectivity.

In the dopamine receptor subgroup, D1-like receptors (D1R and D5R) couple to G_s,_ while D2-like receptors (D2R, D3R and D4R) are recruited to G_i_. The antagonistic regulation of intracellular cAMP levels by D1-like and D2-like receptors reflects their distinct roles in modulating physiological functions. Understanding the functional differences and selective recruitment of G-protein subtypes by D1-like and D2-like receptors provides a foundation for designing drugs that can modulate these receptors in a highly specific and controlled manner, potentially leading to more effective treatments for a range of neuropsychiatric and neurological disorders. Structural comparisons of D1R-G_s_ and D2R-G_i_ structures may provide insight into G_s_ and G_i_ coupling selectivity [140]. In the D1R-G_s_ complex, TM6 moves outward 8.4 Å more than D2R. The larger outward movement of TM6 allows it to accommodate the bulky amino acid on the α5 helix of G_s_, where D2R may form a severe steric clash between TM6 and the α5 helix of G_s_ [140]. Additionally, compared with D2R, TM5 of D1R extends an additional two and a half helix turns on the cytoplasmic side, directly interacting with the Ras domain of G_s_ (Fig. 8b) [140]. This characteristic, where TM5 of the G_s_-coupled receptor is longer than the G_i_-coupled receptor, has been observed in structural comparisons between 5HT_4/6/7_R-G_s_ and 5HT_1/4_R-G_i_ [141].

Furthermore, sequence alignment of TM5 of the G_s_-coupled receptor revealed that the A/V^5.65^ × ^5.69^ motif (x is mostly a hydrophobic residue), which is relatively conserved among D1R, D5R, and β2AR, plays an important role in G_s_ coupling [142, 143]. In the D1R-G_s_ structure, A^5.65^ points toward the hydrophobic pocket composed of L388^G.H5.20^, L394^G.H5.25^ and L395^G.H5.26^, while I^5.69^ forms hydrophobic interactions with the Ras domain of G_s_ (Fig. 8c) [142]. Notably, mutation of A^5.65^ into valine has a lesser impact on the activation potency of dopamine, whereas it significantly decreases with leucine substitution due to the side chain of leucine forming steric clashes with the hydrophobic pocket of G_s_ [142]. In contrast, the corresponding A/V^5.65^ × ^5.69^ motif is L^5.65^xxxR/E^5.69^ (R^5.68^ in D2R), which is also found in other G_i_-coupled receptors, such as δOR, µOR, and κOR (Fig. 8c) [143]. The residue at position 5.69 is mostly a charged residue that differs from D1R. Previous studies have suggested that the C-terminus of TM5 in G_i_-coupled receptors contains charged residues that play a crucial role in G_i_ coupling selectivity. Substitution of x^5.69^ in the A/V^5.65^ × ^5.69^ motif of D1R to a charged residue significantly affects the potency of dopamine [144].

Additionally, the residue at position 34.51 of ICL2 also contributes to G_s_ coupling selectivity [143, 145, 146]. The residue corresponding to position 34.51 in D1R is F129^ICL2^, which interacts with a hydrophobic pocket formed by β1 and β3 strands and the α5 helix of G_s_ (Fig. 7d). Replacing F129^ICL2^ with a small side chain, such as leucine and alanine, deeply influences G_s_ coupling selectivity [143]. A similar pattern can also be observed for β1AR and β2AR [147]. In contrast, the allelic residue in D2R is M140^ICL2,^ which contacts L194^G.S3.01^ and I343^G.H5.15^ of G_i_ through weak hydrophobic interactions (Fig. 8d) [143].

#### G-protein promiscuity and biased signaling pathway of G-protein subtypes

Most GPCRs recruit a specific subtype G-protein to elicit cytoplasmic signal transduction. However, numerous GPCRs bind to diverse G-protein subfamilies, such as CCK1A [148], NTS1R [149], and GPR120. Each of the G-protein subtype signaling pathways may be correlated with distinct physiological or pathological processes. For instance, EP4, a type of prostaglandin E2 (PGE2) receptor, holds therapeutic potential for various conditions, including kidney injury (KI) and X-linked nephrogenic diabetes insipidus (NDI) [150]. Interestingly, the G_s_ signaling pathway of the EP4 receptor has shown beneficial effects on KI and NDI, while the G_i_ signaling pathway can modulate neurotransmitter release and cell migration [151]. Understanding the molecular mechanism for biased G-protein subtype signal transduction can lead to more effective and safer therapeutic interventions for GPCRs.

Recently, the team of Sun Jin-Peng revealed the properties for biased G-protein subtype signal transduction of GPR120. Structural analysis of GPR120 in complex with different G-proteins revealed that the overall architecture of the receptor is equivalent regardless of the G-protein subtype [152]. Among the 24 residues involved in mediating the recruitment of G_i_ and G_q_, 19 residues form conserved interactions with the αN and α5 helices (αH5) of G_i_ and G_q_, whereas 15 residues interact with G_i_ within the 23 residues of GPR120 in contact with G_s_, potentially explaining the G-protein promiscuity of GPR120. The three subtypes of G-proteins (G_i_, G_q_, and G_s_) possess partially distinctive features at the G-protein coupling interface. Through mutation experiments, it has been confirmed that R240^5.71^, S248^ICL3^, or D259^6.30^ in GPR120 is crucial for coupling with G_i_ but does not contribute to G_q_ coupling. The diversity of signaling pathways mediates distinct pathophysiological events.

The GPR120 ligands exhibit different signaling pathways and achieve functional selectivity. Structural analysis of GPR120 in complex with diverse ligands, such as the G_q_-biased ligand TUG891 and unsaturated FAs, reveals different recognition models for ligands in the pocket, operating distinct propagating paths, which may underlie differential G-protein subtype coupling. Within these diverse signaling pathways, conformational locks located at TM3 and TM4 are responsible for connecting the specific π-π interactions in the GPR120 binding pocket and the structural rearrangements coupled to G_s_-protein on the cytoplasmic side. Meanwhile, conformational locks in TM1-TM2 and TM7 are responsible for connecting the ligand pockets with the downstream identification and selective coupling of G_q_ and G_i_. In the G_q_ signaling propagating path, TUG891 forms π-π interactions with the consecutive residues F88^2.53^ and F311^7.43^ in the binding pocket, along with the cytoplasmic side hydrophobic packing between Y227^5.58^ and Y321^7.53^, enabling an outward tilt of TM7 and an inward tilt of TM1 (Fig. 8e). This allows for a tighter insertion of the α5 helix of G_q_, facilitating the crucial cation-π interaction between receptor R136^3.50^ and Y356^G.H5.23^ of G_q_, which is essential for G_q_ bias. In contrast, the bias toward G_s_ is closely related to the propagating path beginning at F211^5.42^, passing through Y165^4.52^- L127^3.41^-I162^4.49^-L158^4.45^-L77^2.42^ and leading to a structural rearrangement of E135^3.49^, ultimately forming a hydrogen bond with Y391^G.H5.23^ of G_s_ (Fig. 8e).

### The arrestin-mediated signaling pathway of GPCRs

In addition to G-protein coupling, GPCRs can recruit arrestin, representing another vital aspect of GPCR signaling. As one of the core regulators of GPCR signal transduction, arrestins participate in regulating GPCR desensitization, internalization, and intracellular transport. Furthermore, they also function as scaffold proteins to activate downstream effector proteins such as mitogen-activated protein kinases (MAPKs), extracellular signal-regulated kinase 1 and 2 (ERK1/2), and Src family tyrosine kinases, thereby playing a crucial role in cell cycle regulation/proliferation and cell survival/apoptosis signal transduction [153]. In addition, recent studies have also identified that the GPCR-βarr1 complex, in addition to serving as a scaffold protein, can also directly activate the protein kinases Src and C-Raf in an allosteric mode [154–156].

There are four subtypes of arrestin: among them, arrestin1 (arr1) and arrestin4 (arr4) are known as visual arrestins and are primarily distributed in the visual sensory system of animals [157]. Arr1 can combine with the light-activated receptor rhodopsin and inhibit its downstream signal transduction, and arr4 can deactivate color opsins [157]. The other two nonvisual arrestins, arrestin2 and arrestin3, are also known as β-arrestin1 (βarr1) and β-arrestin2 (βarr2) [158]. They are widely expressed in various tissues and can be recruited by phosphorylated receptors and subsequently regulate multiple (patho)physiological processes [157].

#### Development progression for GPCR-arrestin structure determination

**Fig. 9:**
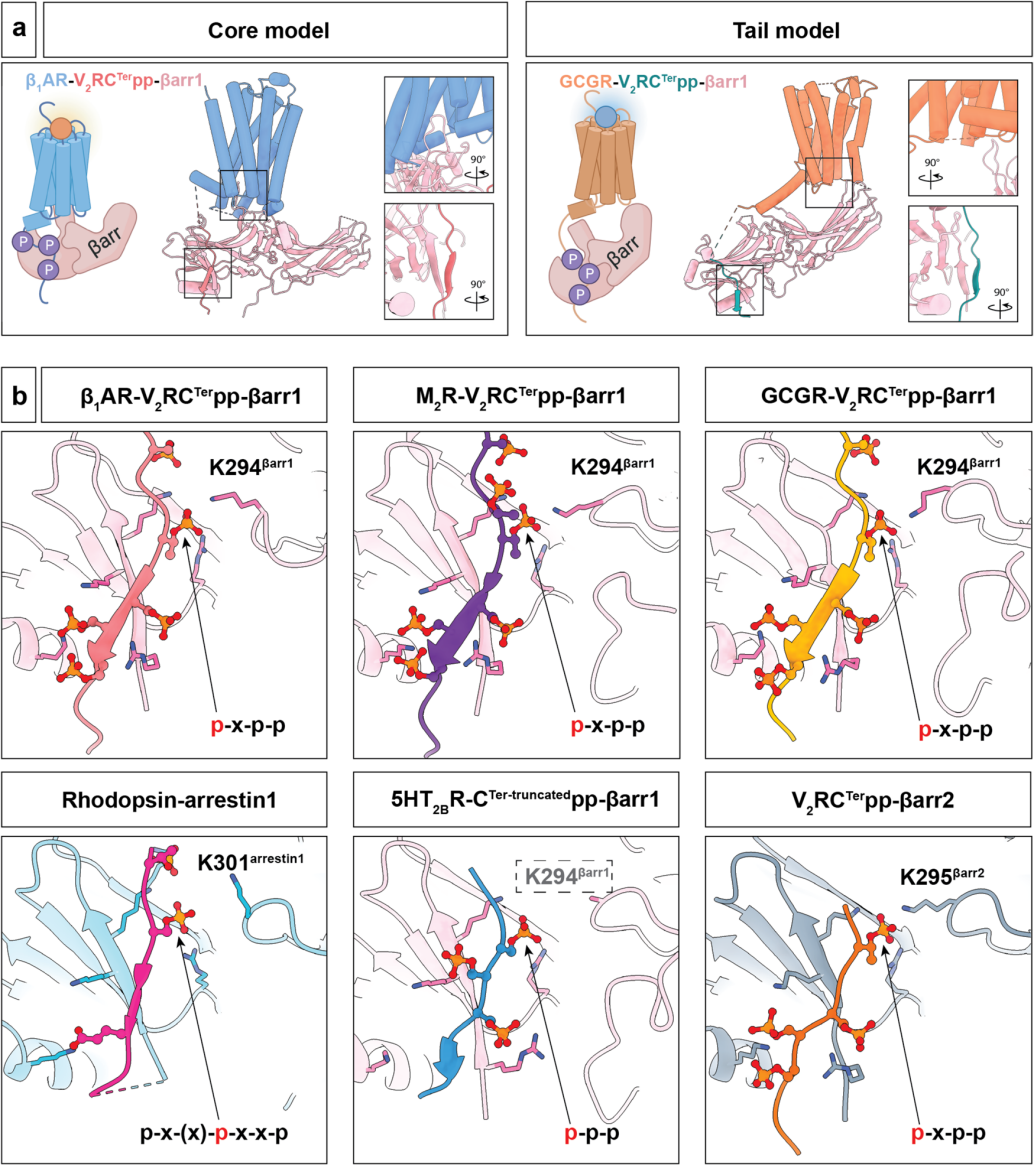
Activation mechanisms of GPCR-arrestin signaling. **a** Two common GPCR-arrestin binding conformations: “Core” mode (e.g., β1AR-V2RC^Ter^pp-βarr1, PDB: 6TKO) and “Tail” mode (e.g., GCGR-V2RC^Ter^pp-βarr1, PDB: 8JRU). **b** Arrestin activation mediated by polar interaction between the key phosphorylation of receptor C-terminus and the lysine in the lariat loop. (β1AR-V2RC^Ter^pp-βarr1, PDB:6TKO; M2R-V2RC^Ter^pp-βarr1, PDB:6U1N; GCGR-V2RC^Ter^pp-βarr1, PDB:8JRU; Rhodopsin-arrestin1, PDB: 4ZWJ; 5HT_2B_R-C^Ter−truncated^pp-βarr1, PDB: 7SRS; V2RC^Ter^pp-βarr2, PDB: 8I10)

Arrestins are composed of the N-domain and the C-domain, each forming a β-stranded sandwich structure connected by a hinge region [158]. With the development of biological techniques, multiple structures of arrestin-bound receptors have been gradually elucidated. In 2013, the crystal structure of βarr1 and the phosphorylated vasopressin-2 receptor carboxyl tail (V2RC) was elucidated, providing a foundation for understanding the activation of βarr1 by phosphorylated receptor tails and the conformational changes that occur after activation (Fig. 9a) [159]. In 2015, a study utilized X-ray free electron laser (XFEL) technology to report the crystal structure of constitutively active human rhodopsin in complex with active mouse visual arrestin, contributing to the understanding of GPCR-mediated arrestin-biased signal transduction [160]. It is acknowledged that GPCRs can bind to arrestin in two ways: one is called the “core” conformation, where the C-terminus of the receptor and the core region of the receptor’s transmembrane domain bind to arrestin together; the other is the “tail” conformation (also acknowledged as the “hanging” mode), where the C-terminus of the receptor binds to arrestin independently [154]. The different conformations indicated above may lead to different receptor signaling pathways. In 2023, the cryo-EM structure of the glucagon receptor (GCGR)-βarr1 complex was elucidated [161]. Functional experiments revealed that the “tail” conformation of GCGR-βarr1 controls the recruitment of βarr1 to the cell membrane and the internalization of GCGR, which is consistent with the previous conclusion (Fig. 9a).

#### Conformation changes of arrestin activation

The phosphorylation of serine and threonine residues in GPCRs, facilitated by GRKs, is typically a prerequisite for binding to arrestins [162, 163]. Upon forming a complex with the phosphopeptide, arrestins undergo a series of conformational transitions, leading to their activation [159, 164]. A central step in this activation is the disruption of the polar core situated in the N-domain. In the context of βarr1 as an illustrative model, the polar core in its apo state harbors a network of highly conserved ionic interactions [159]. These include D26^βarr1^ and R169^βarr1^ in the N-domain, D290^βarr1^ and D297^βarr1^ on the lariat loop within the C-domain, and R393^βarr1^ positioned after the C-terminal strand β20^βarr1^. In concert with these elements, a salt bridge between R25^βarr1^ and E389^βarr1^, coupled with hydrogen bonds uniting strands β1^βarr1^ and β20^βarr1^, reinforces the linkage between the two arrestin domains and stabilizes its quiescent conformation. Upon arrestin activation, the emergence of the phosphopeptide-arrestin complex is characterized by the substitution of arrestin’s strand β20 in its intramolecular β-sheet with β1 by the phosphopeptide [165]. This pivotal intermolecular β-strand exchange results in the release of the entire arrestin C-terminus from its core. Subsequently, conformational changes occur within the arrestin crest, particularly in the lariat, finger, and middle loops, followed by a twist between its N- and C-terminal domains [158].

While the exact mechanism by which phosphopeptide attracts arrestin and displaces the C-terminus of arrestin remains to be determined, it is evident that phosphopeptide binding serves as the initiating force for arrestin activation [166–168]. Several studies have identified unique phosphopeptide recognition motifs across different arrestin isoforms. For instance, the p-×-(×)-p-×-×-p motif was proposed from the rhodopsin-arrestin1 structural analysis [164]. Moreover, recent research has highlighted a p-×-p-p phosphorylation motif in GPCRs (Fig. 9b) [167, 168]. This motif interacts with a spatially organized K-K-R-R-K-K sequence present in the N-domains of both βarr1 and βarr2, indicating a shared activation motif of βarrs. Intriguingly, many GPCRs incorporate the p-×-p-p motif, located either in their C-terminus or ICL3, underscoring its widespread role in facilitating activation [167, 169]. In detail, the initial phosphoresidue of the p-×-p-p motif disrupts existing salt bridges between R169^βarr1^ (R170^βarr2^) and D290^βarr1^/D297^βarr1^ (D291^βarr2^/D298^βarr2^) and establishes new intermolecular salt bridges with R25^βarr1^ (R26^βarr2^), K11^βarr1^ (K12^βarr2^), and K294^βarr1^ (K295^βarr2^), effectively engaging the lariat loop. The interaction between the first phosphoresidue and K294^βarr1^ (K295^βarr2^) represents a primary driving force, drawing the lariat loop closer to the N-domain and inducing a conformational twist in the C-domain. This observation aligns with findings from the recently characterized GCGR(V2RC)-βarrestin1 complex, suggesting a general mechanism of phosphorylation-driven arrestin activation (Fig. 9b) [161]. Notably, in the ACKR3pp-βarr2 complex, while ACKR3pp extends toward the finger loop with its adoption of a p-×-×-p-×-×-p motif rather than p-×-p-p, its pT342 aligns with the initial phosphoresidue in the p-×-p-p motif, hinting at a consistent mode of arrestin activation [170].

Beyond the central role of the 1st phosphoresidue in the motif, other phosphoresidues also significantly influence arrestin signaling. Specifically, the terminal residue of the p-×-p-p motif appears essential for βarr1 recruitment of CCR5, as evidenced by the notable reduction in efficacy when CCR5^T343A^ is introduced in functional assays [168]. Additional phosphorylation sites are postulated to enhance the affinity of GPCR-arrestin interactions and modulate subsequent signaling activities, in line with previous suggestions. It is noteworthy that CCR5pp with only 3 phosphorylated sites of the p-×-p-p motif prompts a similar βarr1-pp-Fab30 population as observed with V2Rpp and the fully phosphorylated CCR5pp [168]. This suggests that the p-×-p-p motif alone is adequate to form a stable and active βarr1-pp-Fab30 complex. Furthermore, it hints at an evolutionary adaptation wherein an increased number of phosphorylation sites can either enhance the GPCR-arrestin complex affinity or act as redundancy in phosphorylation regulation. It is also worth noting that multiple GPCRs exhibit several instances of the p-×-p-p motif in their C-terminus and ICL3, similar to the p-×-(×)-p-×-×-p patterns as previously described [167]. Some receptors also present with an extended ICL3 but have a short or absent C-terminus [157, 171, 172]. The variance in the lengths of the C-terminal tail and ICL3, combined with the density of phosphorylation sites, highlights the inherent structural diversity within the GPCR-arrestin system, which orchestrates their functional versatility (Fig. 9b).

Another GPCR-arrestin binding interface is between the receptor core and the arrestin central crest loops, which represents the “core” mode [157, 160, 173]. In contrast, this interaction is absent in the “tail” mode of GPCR-arrestin engagement. Among the known structures, only the GCGR(V2RC)-βarr1 complex represents the “tail” conformation [161]. The interactions stem from the C-terminus of GCGR, including both helix VIII and the V2R tail, leaving its intracellular pocket vacant. In contrast, previously elucidated GPCR-arrestin complexes predominantly display a core conformation wherein the finger loop of arrestins penetrates into the intracellular pocket of the receptor’s helical bundle [157, 160, 164, 171–175]. In the GCGR(V2RC)-βarr1 complex, the central crest loops of βarr1, including the finger loop, extensively engage with helix VIII of the GCGR. Notably, while the finger loop does not directly interact with the receptor, removing its entire turn region (residues 64-77) of βarr1 significantly diminishes its recruitment. This may result from an altered conformation within the βarr1 central crest or potentially disrupt an alternative arrestin-binding mode, such as the “core” configuration (Fig. 9b) [161, 176].

Investigations on β2AR, V2R, and GCGR indicate that when βarr1 adopts a tail conformation, it predominantly participates in cellular trafficking [177, 178]. Conversely, the desensitization of G-protein activation is uniquely orchestrated by the arrestins engaged with the receptor core. This engagement is indispensable, as spatial hindrance is needed for effectively inhibiting G-protein coupling. In addition, the “tail” conformation plays a pivotal role in maintaining sustained signaling within endosomes, such as the continuous production of second messenger molecules. This is further supported by the demonstrated existence of the G_s_-GCGR-βarr1 megaplex in signaling assays [161]. Such revelations highlight the intricate mechanisms through which arrestin modulates receptor functionality.

#### The barcode hypothesis for arrestin-mediated signal regulation

Previous studies have already found that phosphorylation of GPCRs in different patterns can lead to different arrestin-mediated signaling effects, referred to as the “barcode hypothesis” [179]. In 2020, molecular dynamics (MD) simulations and site-directed spectroscopy were applied to investigate the impact of GPCR phosphorylation patterns on arrestin binding and conformation [180]. The authors found that phosphopeptides with the same number of phosphates can activate arrestin to diverse degrees. Furthermore, the affinity of phosphopeptides to βarr1 depends on the spatial arrangement of phosphorylated residues rather than their quantity. For example, when phosphorylation occurs solely at S350^βarr1^ or T360^βarr1^, the addition of phosphate at S357^βarr1^ significantly reduces arrestin activity. Similarly, when phosphorylation occurs exclusively at S350^βarr1^, the addition of phosphate at S362^βarr1^ significantly decreases binding stability. In addition, the results presented by the site-directed spectroscopy method also indicate that different arrestin structural domains can independently alter conformation. GPCR phosphorylation can affect the conformation of certain arrestin structures without affecting other domains, suggesting that specific conformational changes in arrestin can be induced by specific phosphorylation patterns, thereby exposing certain downstream signal protein binding sites and thus affecting specific downstream effects. Together, these studies reveal the structural basis of the “barcode hypothesis” and highlight its significance in the design of functionally selective GPCR-targeting drugs.

In addition, in the study of the phosphorylation-encoding mechanism of GPCRs, researchers have innovatively proposed the “flute model” theory for receptor phosphorylation [181]. In this article, different phosphorylation barcodes induce distinct structural rearrangements in βarr1, potentially imparting different functions to βarr1 through clathrin, SRC, ERK, or other downstream effector proteins. In 2021, they further analyzed the crystal structure of complexes formed by V2RC with four different phosphorylation patterns and βarr1 [182]. This revealed that a single phosphorylation site defect in GPCR can lead to distinct conformational changes in the distant functional domain of arrestin. Moreover, mutations at different phosphorylation sites in V2RC can result in varying degrees of impact on arrestin recruitment for MEK and c-Raf-1. This study not only reveals the regulatory mechanism of a single phosphorylation site on arrestin function but also discovers the sequential principles in the phosphorylation encoding process, where the binding of phosphorylation sites at certain positions determines whether other positions can bind.

Moreover, studies have also observed a similar degree of desensitization in both wild-type (WT) and GRK (-) (all sites that can be phosphorylated by GRK are mutated) dopamine D2R, suggesting that phosphorylation is not necessary for the arrestin-D2R interaction [183]. However, when detecting the recycling of D2R to the cell surface following agonist-induced endocytosis, the GRK (-) receptor exhibited less recycling than the WT receptor, indicating that phosphorylation can facilitate receptor recycling. While arrestins may not necessarily rely on phosphorylation to exert their functions, phosphorylation remains one of the primary regulatory mechanisms.

### Noncanonical GPCR signal transduction

In addition to the G-protein and arrestin pathways, noncanonical GPCR signaling is also involved in various physiological processes, often in a cell type-specific or context-dependent manner. These pathways are particularly essential in cell proliferation/survival, neurotransmission, immune function, and metabolic regulation. Despite the growing appreciation of the importance of noncanonical GPCR signaling, much remains to be learned about the specific roles of these pathways in physiology and disease. The recent determination of the GPR158-RGS7-Gβ5 complex has provided valuable insights into the operation of noncanonical signaling pathways by GPCRs. GPR158 mainly functions as a homodimer and is distinguished by noncanonical signal transduction pathways [79, 184], which employ G-protein-independent modes. In its ligand-free state, the dimer interface is formed by the apical portion of the cache domain, the extracellular ends of TM4-5 and ECL2, along with the intracellular end of TM3 and ICL2 [185]. This consortium asymmetrically couples with the RGS7-Gβ5 heterodimeric complex [185]. Intriguingly, GPR158 does not directly interact with Gβ5 but establishes direct interactions with RGS7 at two distinct sites [185]. The first interface is formed between the C-terminal coiled-coil configuration (CT-CC) of GPR158 and the DEP-DHEX domain of RGS7, facilitated by amphiphilic interactions. The secondary interface is characterized by the engagement of the intracellular facet of one GPR158 subunit, specifically TM3, TM5, and ICL3, with the DHEX domain of RGS7. This interface overlaps with the interaction domains commonly seen in GPCR-G-protein and β-arrestin complexes [185]. Thus, recruitment of RGS7-Gb5 would preclude GPR158 from interacting with the G-protein, supporting a lack of G-protein activation [185].

In 2023, GPR158 was identified as a metabotropic glycine receptor (mGlyR) [79]. Glycine, when bound to the cache domain, acts as an antagonist to the GPR158-RGS7-Gb5 complex. RGS7-Gb5 is a selective guanosine triphosphatase (GTPase)-activating protein (GAP) for G_i/o_ proteins [79]. Glycine specifically inhibited the GAP activity of RGS7-Gb5 by engaging GPR158 [79]. This, in turn, suppresses the inactivation of Gα_o_, leading to a subsequent reduction in the secondary messenger cAMP, further eliciting a cellular response and regulating neuronal excitability [79]. These findings highlight that GPR158 is no longer an “orphaned” receptor, with implications for targeted drug research and development from its endogenous ligand “glycine”.

### Unveiling the functional and structural characteristics of GRK

G protein-coupled receptor kinases (GRKs) are involved in phosphorylation-dependent or phosphorylation-independent regulation of GPCRs [162, 163, 186], which plays a key role in physiological and pathophysiological processes such as cardiovascular biology, neurodegeneration, and immune response [187, 188]. For instance, overexpression of GRK2 and GRK5 in vivo decreases adrenergic receptor-induced myocardial contractility and cardiac output, whereas inhibition of GRK2, GRK3, and GRK5 counteracts this effect [187, 188]. Phosphorylation of the schizophrenia-associated D3 receptor by GRK2 disrupts the interaction between the receptor and filamin A [188]. High expression of GRK2 and GRK5 in sepsis induces phosphorylation of chemotactic receptors such as CXCR1, thereby inhibiting neutrophil migration [188]. GRK3 inhibits breast cancer metastasis by regulating CXCR4 signaling [187].

#### Subtype selectivity in GRK

The seven GRKs are grouped into the rhodopsin kinase subfamily (GRK1 and GRK7), the β-adrenergic receptor kinase subfamily (GRK2 and GRK3), and the GRK4 subfamily (GRK4, GRK5 and GRK6) [162, 163]. While all GRKs possess conserved sequence characteristics and structural alignments, they employ distinct mechanisms to regulate GPCRs [162, 163, 189]. GRK1/7 and GRK2/3 are activated only by binding to active GPCRs, whereas GRK5/6 also phosphorylates inactive GPCRs [189, 190]. For instance, GRK2 phosphorylates only the C-terminal Ser residue of Neurotensin Receptor 1 (NTSR1) and is agonist dependent, whereas GRK5 phosphorylates NTSR1 intracellular loop 3 and the C-terminal Ser and Thr residues in an activation-independent manner [190]. The order of GRK-mediated GPCR phosphorylation can be barcoded (receptors responding to a specific agonist are phosphorylated at different sites by different GRKs, creating a “barcode”), sequential (a larger number of serine/threonine residues are phosphorylated first), or hierarchical (specific sequences of serines and threonines are preferentially targeted) [187, 188]. Notably, AT1R recruitment of β-arrestin for Ang II binding relies on both GRK2/3 and GRK5/6. However, binding to the β-arrestin-biased ligand TRV027 solely depends on GRK5/6 [191].

#### Advancements in the structural understanding of GPCR-GRK complexes

To date, the structures of rhodopsin-GRK1 and NTSR1-GRK2 have been determined, which revealed that the mode of interaction between GRK and GPCR depends on the activation states of GPCR and GRK [162, 163]. Considering GRK2-NTSR1 as an example, compared to the inactive NTSR1 structure, the cytoplasmic ends of TM5, TM6, and TM7 of NTSR1 are shifted by 4.5 Å, 11.3 Å, and 1.5 Å, respectively, and ICL2 adopts an α-helical structure consistent with an active conformation. The GRK2 structure from the NTSR1 complex, compared with the inactive state, contains an N-terminal helix that is packed onto the kinase domain, has a break in the ionic lock between its RHD from the kinase domain, and adopts a closed conformation in its kinase domain that is in the active state. The GRK2-NTSR1 complex has a major interface consisting of the N-terminal helix of GRK2 that inserts into the open TM6 pocket in a manner that overlaps with the finger loop of arrestin and a minor interface consisting of ICL2 of NTSR1 that interacts with the loop between the N-terminal helix and the RHD [163]. However, it is not ICL2 of rhodopsin but ICL1 and ICL3 that interact with GRK1 in the rhodopsin-GRK1 complex [162]. According to the structure of the GPCR-GRK complex, the extended loop of the intracellular third loop (ICL3) or the extended C-terminal tail of the GPCR reaches the active cleft of GRK, allowing GRK to phosphorylate it [163]. The phosphorylation of the GPCR further facilitates the recruitment of arrestin, which prevents G-protein binding and desensitizes G-protein signaling [162, 163].

Although all GRKs have regulator of G-protein signaling homology (RH) domains, it appears that only the RH structural domain of GRK2/3 binds Gα_q_, whereas the RH structural domains of the other GRKs do not appear to be able to interact with any of the G-proteins because they lack key binding residues [189]. Furthermore, the GRK2/3 subfamily contains a pleckstrin homology (PH) domain that facilitates recruitment to the membrane via interaction with Gβγ subunits [186]. GRK2/3 achieves phosphorylation-independent regulation of GPCRs by binding Gα_q_ and Gβγ, sequestering downstream effectors [163, 189]. For example, GRK2 rapidly and transiently recruits arrestin and induces desensitization without initiating endocytosis by inhibiting G_q_ coupling when µOR and δOR are unphosphorylated [163]. GRK2, by binding Gβγ, inhibits the Gβγ signaling process of adenosine A1, µ-opioid receptor or κ-opioid receptor [189].

GRKs may also facilitate biased signaling through their key role in arrestin recruitment [163, 186]. The allosteric modulator SBI-553 of NTSR1 promotes the binding of GRK2, reshaping the interface in a manner that is compatible with β-arr2 binding but conflicts with Gα_q_ protein binding [163]. D2R may also directly recruit GRK2 to mediate biased signaling of the arrestin-biased agonist UNC9994 [186]. GRK2 activity was needed for receptor phosphorylation and arrestin recruitment in an arrestin-biased D2R mutant unable to bind G-protein [186]. A G-protein-biased D2R mutant deficient in arrestin recruitment also exhibited reduced GRK2 recruitment [186]. The G-protein-biased β2AR mutant Y129A is unable to recruit arrestin due to a lack of phosphorylation by GRKs [186]. In addition, G-protein bias is induced by mutation of M_3_AChR phosphorylation sites [186]. These findings suggest that GRK-mediated phosphorylation may serve as an intervening target to regulate biased signaling in GPCRs.

## GPCR drug discovery

As an important therapeutic target, the low subtype specificity and significant toxic side effects of GPCR ligands limit their therapeutic potential. Key constraints in GPCR drug development include limited drug selectivity, imprecise modulation of receptor signaling pathways, and issues related to tolerance and desensitization.

### Development of GPCR-selective drugs

The pursuit of selective GPCR drugs has garnered significant attention due to its numerous advantages:

#### Enhanced therapeutic efficacy

Selective drugs can precisely target specific GPCR subtypes or signaling pathways, finely modulate GPCR function and effectively intervene in relevant physiological functions or pathological processes.

#### Reduced adverse effects

Drugs that act on multiple GPCRs may induce a range of off-target adverse effects. Selective drugs minimize their impact on other subtype GPCRs, thereby preventing such issues.

#### Enhanced drug safety and predictability

Selective drugs serve as valuable tools in early drug development stages, facilitating the study of GPCR physiological and pathological functions, as well as the understanding of distinct signaling pathways among GPCR subgroups. These insights can inform assessments of pharmacological mechanisms and pharmacokinetic properties, thereby enhancing drug safety and predictability.

The dynamic and plastic properties of the ligand binding pocket of GPCRs present opportunities for identifying new druggable sites, known as extended binding pockets (EBPs). In contrast to the orthosteric binding pocket (OBP), the EBP plays a pivotal role in determining ligand selectivity. In the context of research focused on developing selective drugs targeting the dopamine receptor subfamily, the EBPs for these receptors exhibit distinct characteristics.

The selective agonists SKF83959 and PW0464, which exhibit high affinity for D1R, occupy the EBP composed of extracellular portions of TM2-3 and TM6-7 [143]. In contrast, the D2-like subfamily shares a similar position for the EBP comprised of TM2-3 and ECL1-2 [192–195]. However, despite the common location, the shapes and sizes of these EBPs are distinctive among different receptors within the D2-like subfamily. The unique distinctions in their extracellular binding pocket (EBP) regions enable the selective targeting of each receptor using distinct ligands with specific properties. This selectivity in targeting can have significant implications for drug development and therapeutic interventions related to these receptors.

When designing subtype-selective ligands, it is essential to pay attention to the specific pathway through which the drug enters the receptor, in addition to considering the EBP. For instance, βARs play a crucial role in mediating physiological responses to catecholamines, such as epinephrine and norepinephrine, to regulate cardiovascular, respiratory and metabolic functions. Epinephrine displays equal affinity and occupies an identical orthosteric binding pocket for both β1AR and β2AR. Norepinephrine is slightly smaller than epinephrine, and its binding pockets in β1AR and β2AR are expected to be nearly identical [196]. However, norepinephrine exhibits significantly higher affinity for β1AR than β2AR.

This significant difference in affinity for norepinephrine between β1AR and β2AR can be attributed to their distinct binding kinetics. The binding rate constant (K_on_) of norepinephrine to β1AR is approximately 22-fold higher than that to β2AR [196]. On the other hand, the dissociation rate constant (K_off_) is approximately 1.5-fold higher for β1AR than β2AR. Consequently, the dissociation constant (Kd), which is a measure of binding affinity (Kd = K_off_/K_on_), is lower for norepinephrine to β1AR, suggesting stronger binding affinity [196]. Further analysis revealed that norepinephrine accesses the orthosteric binding pockets of β1AR and β2AR through distinct binding pathways [196]. Key amino acids within these pathways play a pivotal role in influencing the binding rate and affinity of norepinephrine, thereby determining its selectivity. In contrast, although epinephrine also follows different binding pathways to interact with β1AR and β2AR, it contains an additional methyl group in its chemical structure relative to norepinephrine [196]. This methyl group introduces electrostatic property differences, rendering epinephrine nonselective for both receptors [196]. These findings may not only contribute to the design of subtype-selective drugs for βARs but also for other GPCRs.

### The development of biased drugs for GPCRs

In addition to selective ligands, biased ligands are a promising direction in drug design for GPCRs. Biased signaling, also known as functional selectivity or biased agonism, is a phenomenon in which a ligand selectively activates either the G-protein or the β-arrestin pathway of a GPCR, leading to distinct functional outcomes. This concept offers tremendous potential for drug innovation, as it allows for the design of ligands that specifically target one signaling pathway while avoiding the activation of the other. This precision has the potential to reduce undesirable side effects and enhance the desired therapeutic effects. Many GPCRs have exhibited biased signaling, opening the door to the development of drugs that leverage this characteristic to create more targeted and potent therapeutics.

**Fig. 10:**
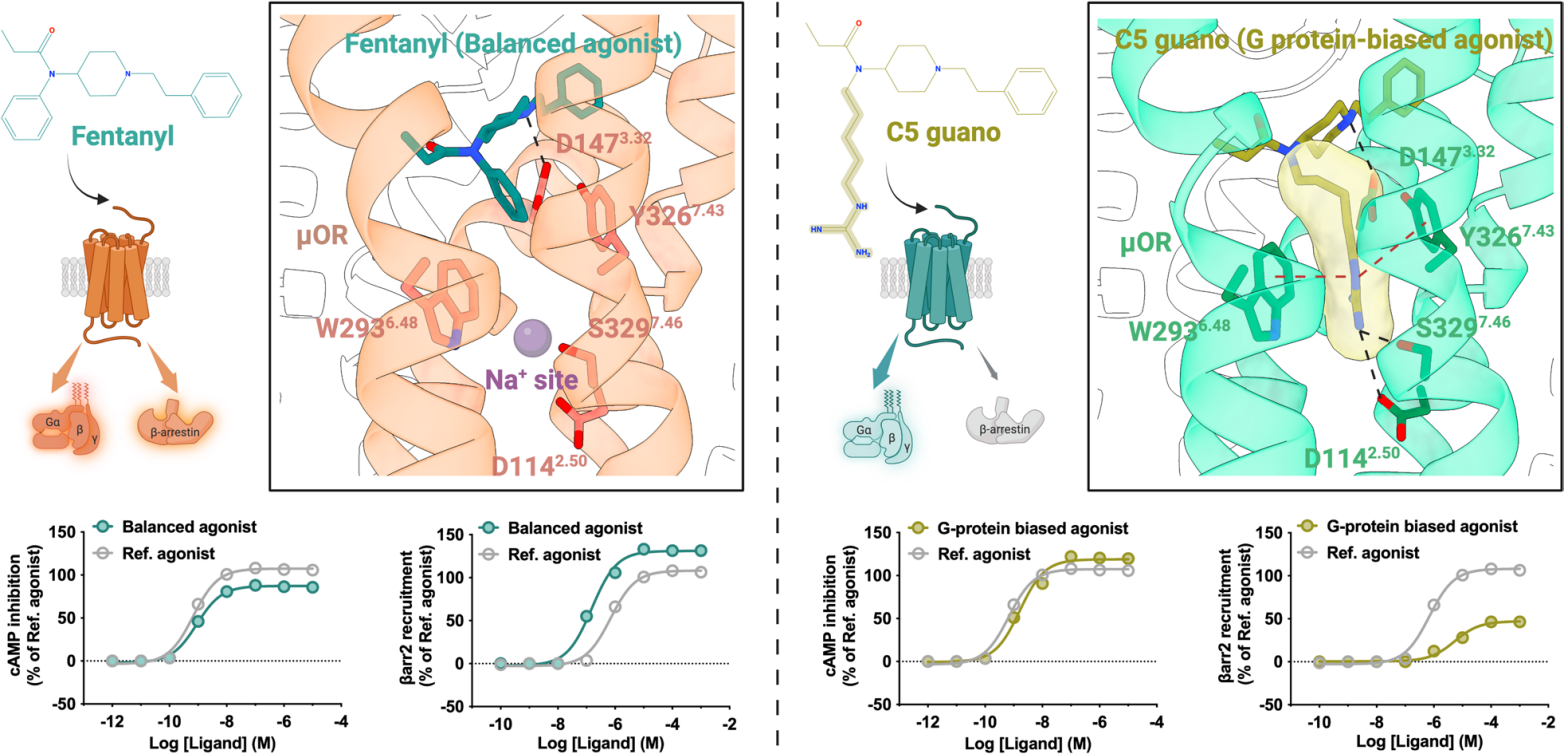
Structural basis of µOR G-protein biased agonism. The interactions between the balanced agonist fentanyl (PDB: 8EF5) or the G-protein-biased ligand C5 guano (PDB: 7U2L) and the involved residues in the µOR orthosteric pocket. The guanidine of C5 guano interacts with the key D^2.50^ in the Na^+^ binding pocket. Salt-bridge interactions and cation-π interactions are represented by black and red dashed lines, respectively. Schematic diagrams of cAMP inhibition and β-arrestin-2 recruitment on µOR with balanced or G-protein biased ligands are displayed compared with the reference ligand

Several mechanisms have been identified that facilitate biased signaling. Investigation of C5aR1 with the G-protein-biased agonist BM213 has pinpointed the binding pocket and “IWI” motif formed by TM2-ECL1-TM3 as playing an essential role in biased signaling, providing insights into the design of C5aR1-biased molecules [197]. The guanidine of the G-protein-biased bitopic ligand C5 (or C6) guano interacts with the key Asp^2.50^ in the sodium ion-binding pocket of µOR, which can reduce or even abolish β-arrestin-1 and β-arrestin-2 recruitment compared with the parent ligand fentanyl (Fig. 10) [7].

In 2020, oliceridine, which is a G-protein-biased agonist for the µ-opioid receptor (µOR), was approved for the management of acute severe pain in adults where alternative treatments fall short. By specifically triggering the G_i_ signaling pathway, oliceridine aims to achieve effective pain relief while minimizing typical side effects, such as respiratory depression and constipation, which are often linked to the activation of the β-arrestin pathway [198]. Additional G-protein-biased µOR agonists, such as PZM21, have demonstrated better analgesic effects and reduced side effects in preclinical pain studies [199]. In addition, several β-arrestin-biased drugs have been identified and studied. Carvedilol, a β-arrestin-biased agonist of β1AR and β2AR, has been shown to counteract the harmful effects of G-protein-mediated catecholamines while promoting β-arrestin-driven cell survival pathways. It is currently further approved for the management of heart failure [200]. Another example, TRV120027, is a potent β-arrestin-biased ligand for AT1R. It holds the potential to enhance cardiac contractility through β-arrestin pathways and maintain cardiac stroke volume, making it a candidate for acute heart failure treatment [201]. However, while these effects were observed in mouse studies, human clinical trials have yet to meet the set clinical endpoints.

Beyond ligand-induced biased signaling, certain receptors also intrinsically exhibit bias during their evolutionary development. For instance, C5aR2 lacks functional G-protein coupling but demonstrates robust β-arrestin recruitment [202]. Sequence alignment indicates that the residues within the intracellular pocket of C5aR2, which are associated with G-protein coupling, are alanine, differing from typical class A GPCRs with G-protein signaling. This distinction might impede G-protein binding, partially explaining why C5aR2 predominantly mediates β-arrestin signaling.

The current therapeutic landscape boasts a range of biased ligands tailored for specific disease treatments. However, certain receptors, such as GPR120, present an intricate picture. GPR120 can engage multiple G-protein subtypes (G_q_/G_i_/G_s_) and β-arrestin1/2, thereby orchestrating a variety of downstream signaling pathways [152]. Thus, the focus should shift toward creating ligands that show a distinct preference for specific G-protein or β-arrestin subtypes. Such innovations could lead to more nuanced control of signaling pathways, offering potential breakthroughs in precision medicine and novel therapeutic strategies. To achieve this, additional structures of biased ligand-GPCR complexes are needed, which will shed light on the mechanisms of biased signaling and offer a refined molecular foundation for drug design.

### Allosteric pharmacology of GPCR and drug discovery

The majority of FDA-approved GPCR drugs on the market target the orthosteric site, which is the same site as the endogenous ligand binding pocket [203]. Although the success of drugs targeting GPCRs has been proven, it is still difficult to design selective ligands or drug candidates for individual GPCRs owing to the high conservation of orthosteric sites among subgroups. Thus, the development of new approaches/strategies for discovering therapeutic agents is imperative. Allosteric modulators, which bind to distinct sites from the orthosteric pocket, have emerged as a promising direction in GPCR drug discovery. They offer unique properties and the potential for increased selectivity. To date, 31 allosteric drugs have entered clinical phases, with six of them receiving FDA approval (Table 1). For instance, Avacopan, a NAM for C5aR, was approved to treat ANCA-associated vasculitis, which is an autoimmune disease, by inhibiting the binding of C5a, thus reducing the inflammatory response and improving autoimmune disease symptoms [204]. Moreover, cinacalcet, which is primarily used to treat hypercalcemia by regulating the secretion of parathyroid hormone to reduce the level of blood calcium, is a PAM for CaSR [205–207].

Allosteric modulators offer a valuable alternative in GPCR drug development, allowing for the development of more selective and precise therapeutic agents, which can enhance treatment efficacy while minimizing off-target effects.

**Table 1:** The allosteric drugs for GPCRs

Drugs	Target	Action	Phase	Indications	References
**Avacopan**	C5aR1	NAM	Approved	Anti-neutrophil cytoplasmic autoantibodies-associated vasculitis	[208]
**Cinacalcet**	CaSR	PAM	Approved	Hyperparathyroidism and calciphylaxis	[205–207]
**Ticagrelor**	P2Y_12_	NAM	Approved	Prevention of thrombosis	[209]
**Ivermectin**	GABA_B_	PAM	Approved	Parasitic roundworm infections	[210]
**ATx-201**	NPY4	PAM	Approved	Viral and bacterial infections;Atopic dermatitis; Cancer;Rheumatoid arthritis;	[211]
**Maraviroc**	CCR5	NAM	Phase III	HIV infection	[212]
**Vercirnon**	CCR9	NAM	Phase III	Inflammatory bowel disease	[213]
**BMS-986,165**	mGluR4	Unclear	Phase III	Plaque psoriasis; Psoriatic arthritis;Crohn’s disease; Systemic lupus erythematosus	Allosteric database
**Mavoglurant**	mGluR5	NAM	Phase III	Fragile X syndrome	[214]
**ADX-48,621**	mGluR5	NAM	Phase III	Parkinson’s disease levodopainduced dyskinesia	[215]
**Basimglurant**	mGluR5	NAM	Phase III	Fragile X syndrome	[216]
**ASP-4345**	D1R	PAM	Phase II	Schizophrenia; Cognitive disorders	[217]
**LY-315,402**	D1R	PAM	Phase II	Dementia; Parkinson	[218]
**ADX-10,059**	mGluR5	NAM	Phase II	Reflux, Gastroesophageal migraines	[219]
**DT-1687**	mGluR4	PAM	Phase II	Parkinson’s disease	Allosteric database
**AZD-8529**	mGluR2	PAM	Phase II	Smoking cessation therapy; Schizophrenia	[220]
**ADX-71,149**	mGluR2	PAM	Phase II	Epilepsy; Anxiety disorder; Schizophrenia	[83, 221]
**MK-7622**	M1	PAM	Phase II	Pain; Schizophrenia; Sleep disorder; Dementia, Alzheimer’s type	[222–224]
**ASP-8302**	M3	PAM	Phase II	Detrusor underactivity (Underactive bladder)	[225]
**Emraclidine**	M4	PAM	Phase II	Schizophrenia	[226]
**T-62**	A_1_AR	PAM	Phase II	Neuropathic pain; Postherpetic neuralgia (PHN)	[227]
**HTL-14,242**	mGluR5	NAM	Phase I	Neurological disorders; Psychiatric disorders	[228]
**RG-7342**	mGluR5	PAM	Phase I	Schizophrenia	Allosteric database
**RGH-618**	mGluR5	NAM	Phase I	Anxiety disorder	Allosteric database
^**(11 C)**^ **JNJ-42,491,293**	mGluR2	PAM	Phase I	Diagnostics	[229]
**JNJ-55,375,515**	mGluR2	NAM	Phase I	Cognitive disorders; Psychosis	Allosteric database
**TAK-071**	M1	PAM	Phase I	Lewy body dementia; Neurological Disorders; Dementia;Alzheimer’s type	[230]
**VU-319**	M1	PAM	Phase I	Pain; Sleep disorder;Dementia; Alzheimer’s type	Allosteric database
^**(11 C)**^ **MK-6884**	M4	PAM	Phase I	Dementia, Alzheimer’s type	[231]
**ODM-106**	GABA_B_	PAM	Phase I	Essential tremor	Allosteric database
**JNJ-2463**	CB1	NAM	Phase I	Nonalcoholic steatohepatitis; Nephropathy, diabetic; Nonalcoholic fatty liver disease (NAFLD); Fibrosis; Metabolic Diseases	[232]

#### Classification of GPCR allosteric modulators

**Fig. 11:**
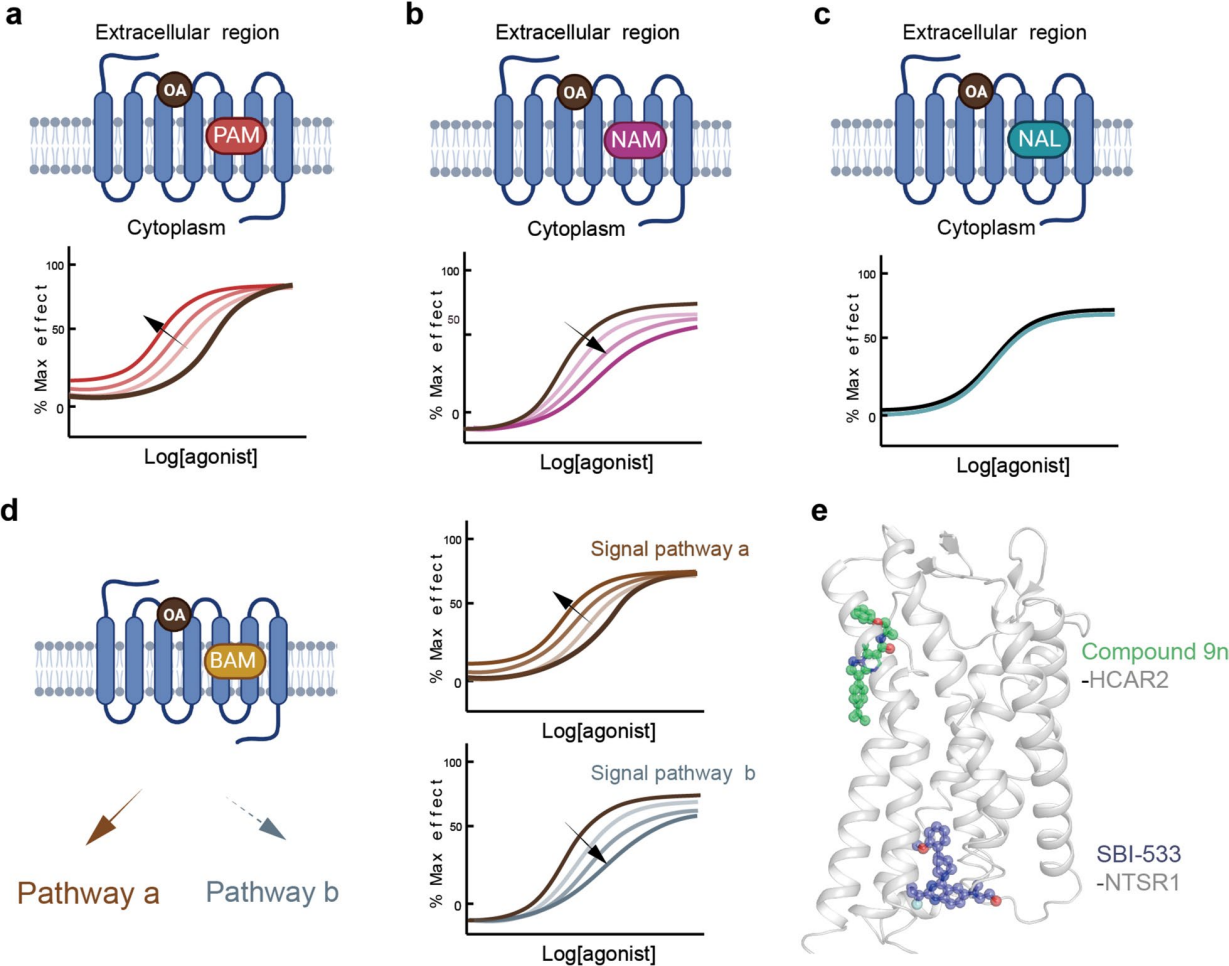
Effects of allosteric modulators on receptor signaling. PAMs (**a**) and NAMs (**b**) modulate the effect of orthosteric agonists, while NALs (**c**) have no influence on receptor signaling mediated by orthosteric agonists. BAMs (**d**) potentiate signaling pathway a while inhibiting signaling pathway b of the receptor. The dose-dependence curves (below) display the effect of orthosteric agonists in the presence of allosteric modulations. The color saturation indicates the concentrations of allosteric modulators. OA: orthosteric agonist. **e** The binding sites of BAMs in receptors. SBI-553 (blue) binds to the pocket located at an intracellular region composed of TM6-7 and H8 of NTSR1 (PDB: 8FN0), while compound 9n sits in the upper portion between TM5-6 and ECL2 of HCAR2 (PDB: 8JII)

GPCRs have multiple functionally distinct conformation states that display varying affinities for orthosteric ligands and different abilities to engage effectors: G-proteins, arrestins and other signaling molecules. Allosteric modulators stabilize the receptor in a specific conformational state and thus modulate the potency of signal transduction [233, 234]. According to the effect on orthosteric ligand, allosteric ligands can be classified into three groups: positive allosteric modulator (PAM), negative allosteric modulator (NAM) and neutral allosteric ligand (NAL) [235, 236]. PAMs and NAMs can modulate the affinity of orthosteric ligands or affect the intrinsic efficacy of orthosteric agonists (OAs). PAMs enhance signaling by increasing the affinity or efficiency of OAs (Fig. 11a), while NAMs do the opposite (Fig. 11b). NAL can bind to the allosteric site but has no effect on signaling (Fig. 11c).

The diverse effects of allosteric modulators on receptor signal transduction can be described and quantified using the allosteric ternary-complex model (ATCM) [237–240]. This model takes into account the interactions among the receptor, orthosteric agonist (OA), allosteric modulator, and effector protein to determine the direction and magnitude of allosterism. The model employs cooperativity factors to govern these interactions.

In the operational model of ATCM:

The modulation of binding affinity is regulated by the cooperativity factor (α). When α > 1, it indicates positive cooperativity, enhancing the affinity of the allosteric agonist (OA) for the receptor. When 0 < α < 1, it denotes negative cooperativity, reducing the affinity of OA for the receptor. A value of α = 1 represents neutral cooperativity, indicating no effect on the affinity of OA for the receptor.

The modulation of OA signaling efficacy is controlled by the cooperativity factor (β). A value of β > 1 signifies positive cooperativity, enhancing the signaling effect of OA. When 0 < β < 1, it indicates negative cooperativity, diminishing OA’s signaling effect. A value of β = 1 signifies no effect on OA signaling.

These cooperativity factors, α and β, provide a quantitative framework for understanding how allosteric modulators influence receptor binding and signaling in the allosteric ternary complex model.

#### Advantages of allosteric pharmacology

The complexity and unique characteristics of allosteric modulation provide the predicted advantages over orthosteric ligands for GPCR drug discovery.

##### Selectivity

One of the primary motivations behind focusing on allosteric compounds in GPCR drug discovery is the hope of developing subtype-selective ligands for specific receptor groups. This is particularly relevant when traditional orthosteric strategies face challenges due to the high similarity in the orthosteric pocket among receptor subtypes. For instance, the high conservation of the orthosteric pocket among cannabinoid receptors implies that it is impossible to design selective orthosteric ligands for CB1 or CB2. Surprisingly, ORG27569, only performed as a NAM for CB1, binds to CB1 in a unique site that is different from CB2 [110, 241]. Meanwhile, ZCZ011, acting as a PAM, displays high subtype selectivity for CB1 [242, 243].

It often appears to be the case that designing a selective allosteric modulator is comparatively easier than developing selective orthosteric ligands. Many allosteric modulators have been identified through screening processes and have shown high subtype selectivity without the need for extensive optimization efforts [244]. This has made allosteric modulation a valuable approach in achieving selectivity for specific GPCR subtypes in drug discovery.

##### Allosteric agonist and ago-PAMs

The classic allosteric modulators, often referred to as pure allosteric modulators, are compounds that exert their allosteric actions only in the presence of OAs [245, 246]. This characteristic suggests several advantages for allosteric modulators, including spatial and temporal specificity. In tissues where the endogenous ligand is present at higher levels, allosteric drugs can exhibit greater sensitivity and efficacy in modulating the receptor’s activity. This sensitivity is linked to the presence of OA. Additionally, the dependence of pure allosteric modulators on the presence of the endogenous ligand results in a ceiling effect for allosterism. This means that even if a higher dose of the allosteric modulator is administered, the response will eventually reach a maximum level, increasing the safety margin of the candidate agent in overdose situations [244].

In addition to pure allosteric modulators, there are also agonist-PAMs (ago-PAMs). These ligands have the intrinsic ability to activate receptor signaling in the absence of an agonist while simultaneously performing the PAM effect [47, 247]. For mutated GPCRs that have lost the ability to bind endogenous ligands, ago-PAMs can act as supplements to restore or partially restore GPCR function [248]. Furthermore, in cases where endogenous ligand levels are low, ago-PAMs can enhance receptor signaling sufficiently due to their high cooperativity and intrinsic activity. This property makes them valuable in situations where endogenous ligands are limited or compromised.

##### Biased activation

GPCRs are dynamic and interact with various effectors, including G-proteins (G_s_, G_i/o_, G_11/q_, and G_12/13_) and arrestins (e.g., β-arrestin1 and β-arrestin2), to mediate distinct physiological effects. Biased ligands are compounds that selectively stimulate specific therapeutic pathways while avoiding unwanted on-target side effects [249]. In addition to biased orthosteric agonists, there are also biased allosteric modulators (BAMs) that can trigger biased signal transduction of GPCRs [249, 250]. BAMs offer the advantage of positive PAMs when combined with orthosteric ligands, allowing for fine-tuned and selective modulation of GPCR signaling pathways (Fig. 11d) [249]. This approach holds promise for the development of more precise and effective therapeutics targeting GPCRs.

#### Biased allosteric pharmacology of GPCR

The binding sites of BAMs (biased allosteric modulators) in GPCRs can vary and are distributed throughout different regions of the receptor (Fig. 11d), including the extracellular region, transmembrane domain (TMD), and cytoplasmic region. These binding sites play a crucial role in allosteric modulation and influence the receptor’s interaction with various signaling pathways. Here are some recent examples of BAM binding sites in GPCRs:

##### Neurotensin receptor (NTSR1)

 A BAM called SBI-553 binds to NTSR1 at an intracellular site that is composed of transmembrane helices TM6 and TM7, as well as helix H8 [149]. The binding of SBI-553 to this unique pocket causes remodeling of the interface between NTSR1 and the Gα protein. This rearrangement affects the conformation of the Gα protein, particularly the wavy hook and α5 helix, which are critical for determining G-protein subgroup selectivity. As a negative allosteric modulator (NAM) for the NTSR G-protein signaling pathway, SBI-553 displays complex allosteric effects with different Gα subtypes (Fig. 11e).

##### Hydroxycarboxylic acid receptor 2 (HCAR2)

Compound 9n is a BAM that binds to HCAR2 [251, 252]. Its binding site is located in the upper half of transmembrane helices TM5 and TM6, as well as extracellular loop 2 (ECL2). This unique binding site contributes to distinct allosteric effects on HCAR2 signaling through both G-proteins and β-arrestin (Fig. 11e).

These structural determinants of GPCRs with allosteric modulators, especially BAMs, are essential for understanding the molecular mechanisms of biased allosteric modulation and advancing the development of biased allosteric pharmacology and biased allosteric drugs for GPCRs. They provide valuable insights into how BAMs can influence receptor conformation and signaling pathways, ultimately aiding in drug design and therapeutic development.

### Poly-pharmacology of GPCR and drug discovery

The drug development strategies mentioned above, including selective ligands, biased ligands, and allosteric ligands, typically focus on targeting specific GPCRs. However, the landscape of drug development is undergoing a paradigm shift, with an increasing emphasis on multitarget drugs. Poly-pharmacology, which refers to drugs that interact with multiple targets, is attributed to complicated biological pathways and consequently to multiple effects [253, 254]. GPCRs frequently share similar structural frameworks, and the key residues within their binding pockets exhibit certain conservation, making them attractive candidates for multitarget drug development [255]. Notably, the interplay of a drug molecule with multiple targets can be a double-edged sword [256]. While it can lead to beneficial outcomes, especially in drug repurposing, it can also result in detrimental off-target effects. Therefore, comprehensive polypharmacological analysis is indispensable in drug development for a holistic understanding of both the desired and potential adverse effects. Here, our focus primarily rests on drugs targeting GPCRs that exhibit therapeutic effects by engaging multiple beneficial targets, sidelining those with predominant side effects.

In the realm of multipharmacological drugs targeting GPCR subfamilies, dual agonists and antagonists often offer enhanced therapeutic outcomes compared with single-target alternatives. A case in point is tirzepatide, a fatty-acid-modified polypeptide. It mirrors the behavior of native GIP at the gastric inhibitory polypeptide receptor (GIPR) and simultaneously showcases a preference for G-protein signaling at GLP-1R [257]. The molecular basis underpinning tirzepatide’s dual agonism has been recently elucidated [258]. Similar to native ligands, tirzepatide adopts an α-helical structure with its N-terminus deeply embedded within the transmembrane core of both receptors. However, a notably compact tirzepatide-GIPR complex has been observed. Specifically, the strong interactions between Tyr1^Tzp^ and other residues within the GIPR core enable tirzepatide to accept fatty acid modifications, thereby achieving an affinity comparable to GIP. In contrast, its high-affinity interaction with the extracellular domain of GLP-1R, combined with the reduced stability from Tyr1^Tzp^ and the lipid moiety, promotes biased signaling and diminishes receptor desensitization. Such bias potentially amplifies the effectiveness of tirzepatide in managing glucose levels and body weight in type 2 diabetes mellitus patients [259, 260]. Furthermore, the therapeutic prospects of tirzepatide extend to conditions such as obesity and nonalcoholic steatohepatitis (NASH), underscoring its versatility in addressing multifaceted diseases [261, 262]. In the context of dual antagonists, bosentan, a compound that concurrently targets both endothelin receptor type A (ETAR) and ETBR, has been approved for addressing pulmonary hypertension [263]. Its interaction with ETAR curtails vasoconstriction, while engagement with ETBR impedes bronchoconstriction [264]. The therapeutic objective is to counteract these constraining effects, facilitating the relaxation of the pulmonary vasculature and thereby attenuating pulmonary pressures and resistance.

While many drugs typically engage in similar binding patterns within the orthosteric pockets of receptors, there are multitarget drugs that operate through diverse pharmacophores. A case in point is sparsentan. This dual antagonist selectively targets both ETAR and AT1R [265]. It is noteworthy for its high affinity for both receptors and was designed by integrating functional structural elements from two separate antagonists, irbesartan (specific to AT1R) and biphenylsulfonamide (specific to ETAR) (Table 2) [266, 267]. This approach epitomizes another dimension in the poly-pharmacological drug design paradigm.

Certain dual-target drugs possess the capability to interact with both GPCR and non-GPCR targets simultaneously. Roluperidone serves as a notable example. As a dual antagonist, it targets the 5HT_2A_R and σ2 receptors [268]. Clinical trials suggest that roluperidone has therapeutic potential in addressing the negative symptoms associated with schizophrenia [269]. This reflects the extensive nature of polypharmacology and shows the potential for drug possibilities across different types of targets.

When considering three or more targeted drugs of GPCR, it emerges passively during the investigation of the pharmacological properties of the drugs. The aminergic receptors, notably the 5-HT receptors, dopaminergic receptors, and adrenoceptors, stand out due to their ligand promiscuity, shared binding modes, and orthosteric pocket similarities [270]. For instance, ergotamine for migraine treatment has broad interactions with multiple receptors and usually causes serious side effects [271, 272]. As the understanding of the molecular mechanisms of the ligand-receptor recognition and downstream signaling initiation has deepened, a few compounds have been designed to specifically target therapeutic receptors or signaling pathways. Drugs such as brexpiprazole and cariprazine exemplify this approach [273]. They act as 5HT_2A_R antagonists while concurrently serving as agonists for 5HT_1A_R and D2R, primarily catering to the treatment of psychiatric conditions.

Another promising frontier in multitarget drug development targets the GLP-1-like receptor subfamily. Prominent examples include the triagonists Retatrutide, HM15211, and MAR423 [274–276]. Taking retatrutide as an illustrative case, it was derived from the GIP architecture, not only mirroring tirzepatide’s dual agonist effects but also introducing enhanced glucagon receptor activation. As of now, Retatrutide is undergoing several phase 3 clinical trials, primarily tending to address cardiovascular and metabolic endocrinological disorders [277, 278].

In summary, the advent of multitarget drugs, derived from a deeper understanding of complicated biological processes, offers a promising avenue for treating a myriad of diseases more holistically and effectively. While the individual activities of multitarget drugs might be subdued compared to their single-target counterparts, their ability to synergistically modulate interconnected disease targets renders them especially advantageous [279]. Such an approach is particularly beneficial for diseases with multiple etiologies, including malignancies, cardiovascular diseases, neurodegenerative disorders, and autoimmune diseases, where poly-pharmacological drugs hold immense prospective value. A list of approved therapeutic polypharmacological agents is shown in Table 2.

**Table 2:** FDA-approved agents targeting GPCRs with polypharmacological characteristics (2010–2023)

First Approval	Drug	Modes of Action	Indications	References
2023	Sparsentan	Antagonist of AT1R and ETAR	Immunoglobulin A Nephropathy	[280]
2022	Tirzepatide	Agonist of GIPR and GLP-1R	Type 2 Diabetes	[281]
2020	Bencycloquidium Bromide	Antagonist of M1 and M3	Allergic Rhinitis	[282, 283]
2020	Ozanimod	Agonist of S1PRs	Crohn’s Disease, Relapsing-Remitting Multiple Sclerosis, Ulcerative Colitis. Multiple Sclerosis	[284–286]
2020	Fenfluramine	Agonist of 5HT_1_Rs and 5HT_2_Rs	Lennox-Gastaut Syndrome, Dravet Syndrome, CDKL5 Deficiency, Seizures	[287–294]
2019	Siponimod	Agonist of S1PR1 and S1PR3-5	Secondary Progressive Multiple Sclerosis, Multiple Sclerosis	[286, 295–299]
2019	Lumateperone	Antagonist of 5HT_2_Rand D2R	Depression, Schizophrenia	[300, 301]
2018	Revefenacin	Antagonist of muscarinic receptors	Chronic Obstructive Pulmonary Disease	[302]
2017	Dinalbuphine Sebacate	Agonist of κOR; Antagonist of µOR	Pain	
2015	Aripiprazole Lauroxil	Agonist of D2R, 5HT_1_Rs, 5HT_2_Rs, and 5HT_7_R; Antagonist of H1R, D4R, and 5HT_6_R	Schizophrenia	[303–308]
2015	Brexpiprazole	Agonist of 5HT_1_R and D2R	Agitation, Severe Depressive Disorder, Schizophrenia	[309, 310]
2015	Cariprazine	Agonist of 5HT_1_R, D2R and D3R; Antagonist of 5HT_2_R	Severe Depressive Disorder, Bipolar Disorder And Related Disorders, Bipolar 1 Disorder, Schizophrenia	[311–313]
2015	Eluxadoline	Agonist of µOR and κOR; Antagonist of δOR	Diarrhea-Type Irritable Bowel Syndrome, Irritable Bowel Syndrome (IBS)	[314]
2015	Flibanserin	Agonist of 5HT_1_R; Antagonist of 5HT_2_R and D4R	Psychosexual Dysfunction	[315]
2013	Vortioxetine	Agonist of 5HT_1A - B_R; Antagonist of 5HT_1D_R, 5HT_2_R, 5HT_3_R and 5HT_7_R	Severe Depressive Disorder, Depressive	[315]
2013	Macitentan	Antagonist of ETAR and ETBR	Connective Tissue Disease, Pulmonary Hypertension	[316]
2012	Loxapine	Antagonist of H1R, 5HT_2_Rs, 5HT_6_R, 5HT_7_R, and D2R-D4R	Bipolar Disorder, Bipolar 1 Disorder, Schizophrenia	[304, 317–323]
2011	Motilitone	Agonist of 5HT_4_R; Antagonist of 5HT_3_R and D2R	Dyspepsia	
2010	Lurasidone	Agonist of 5HT_1_R; Antagonist of 5HT_2_R, 5HT_7_R, D2R, Α2AR and Α2CR	Bipolar Disorder, Bipolar 1 Disorder, Schizophrenia	[324]
2010	Fingolimod	Agonist of S1PR1 and S1PR5; Modulator of S1PR3 and S1PR4	Relapsing-Remitting Multiple Sclerosis, Multiple Sclerosis, The Relapsing Multiple Sclerosis	[325]
2010	Buprenorphine	Agonist of µOR; Antagonist of κOR	Opium Dependence, Opioid-Related Disorder, Pain	[326, 327]

The drugs listed above were identified from databases such as guide to pharmacology, Drug Bank and zhihuiya new drug repository.

### Antibody drug development for GPCRs

Compared to conventional chemical drugs, antibody-based therapies for GPCRs are preferred by major domestic and international pharmaceutical companies due to their unique properties, which include specificity, high affinity, and longer serum half-life. GPCR antibody drugs can be categorized into three main groups:

#### Anti-receptor antibody

These antibodies directly target GPCRs themselves, interfering with receptor function or signaling pathways. Currently, there are two FDA-approved antibody drugs targeting GPCRs. Erenumab, a selective monoclonal antibody designed to target the calcitonin gene-related peptide type 1 receptor (CGRPR), received FDA approval in 2018 for the treatment of migraines [328]. Mogamulizumab, a humanized monoclonal antibody for CCR4, is employed in the treatment of two rare cutaneous T-cell lymphomas (Table 3) [329].

#### Anti-ligand antibody

These antibodies are designed to block or neutralize ligands (molecules that activate GPCRs) and can indirectly modulate GPCR activity. For instance, anti-calcitonin gene-related peptide (anti-CGRP) monoclonal antibodies such as fremanezumab, ellipsumab-jjmr, and galcanezumab have shown promise in the treatment of migraines [330]. They selectively target both the α and β subunits of human CGRP, leading to the blockade of CGRPP activation. In 2023, a significant milestone was achieved with the FDA approval of Veopoz (pozelimab) [331]. Veopoz is a fully human monoclonal IgG4 antibody with high binding affinity for wild-type and variant human complement C5 proteins and effectively inhibits the activity of complement factor C5 for therapeutic purposes. Veopoz represents a groundbreaking therapy, being the first FDA-approved treatment for CD55-deficient protein-losing enteropathy (CHAPLE) in adults and pediatric patients over the age of 1 year. This achievement highlights the potential of monoclonal antibodies in addressing rare and complex medical conditions.

#### Antibody-drug conjugates (ADCs) for GPCRs

ADCs are a class of targeted drugs that combine monoclonal antibodies with cytotoxic drugs via linkers. These ADCs leverage the targeting capability of antibodies to deliver toxic drugs specifically to cells expressing the target GPCR, reducing drug toxicity while maintaining therapeutic efficacy. Globally, there are 19 ADC candidate drugs designed to target GPCRs. Among these, 4 are in clinical phase I or II, 6 are in preclinical stages, 2 are in drug discovery phases, and 7 are not in active development. These ADCs are designed to target a range of GPCRs, including CXCR4, CCR5, CCR3, C5AR1, LGR4 and others, presenting a diverse set of potential therapeutic options. Furthermore, significant progress has been made in the development of ADC drugs targeting orphan GPCRs. A noteworthy achievement in this context is the recent FDA approval of the world’s first bispecific antibody targeting GPRC5D/CD3 for the treatment of relapsed/refractory multiple myeloma. This groundbreaking therapy is specifically designed to target the orphan class C GPCR GPRC5D, which exhibits high expression levels in affected patients. This achievement underscores the potential of ADCs in addressing previously untargeted or challenging medical conditions.

The development landscape for GPCR antibody drugs has expanded significantly, encompassing various innovative approaches and combinations to enhance therapeutic efficacy, including bispecific antibodies, nanobodies, and combinations with other therapies such as CAR-T (chimeric antigen receptor T cell), checkpoint inhibitors, chemotherapy drugs, and cell therapies. The growing number of projects in the global research and development pipeline, along with the successful entry of GPCR antibodies into clinical development over the past decade, underscores the importance of targeting GPCRs with monoclonal antibodies in pharmaceutical research. This expanding landscape reflects the pharmaceutical industry’s commitment to exploring innovative approaches to address a wide range of medical conditions and advance the field of GPCR-based therapeutics. A list of the antibody drugs for GPCRs is shown in Table 3.

**Table 3:** The antibody drugs for GPCRs

Drugs	Target	Phase	Indications	References
**Erenumab**	CGRP	Approved	Migraine,Temporomandibular joint dysfunction syndrome, Headache	[332]
**Mogamulizumab**	CCR4	Approved	Cancer, Adult T-cell Leukemia/Lymphoma (ATLL), Cutaneous T-cell Lymphoma (CTCL)	[333–336]
**Leronlimab**	CCR5	Phase III	COVID-19 Pneumonia, Metastatic microsatellite-stabilized colorectal cancer, Nonalcoholic steatohepatitis	[337]
**Talquetamab**	GPRC5D	Phase III	Multiple Myeloma, Plasma cell myeloma refractory, Recurrent Multiple Myeloma	[338, 339]
**REMD-477**	GCGR	Phase II	Type 1 diabetes, Type 2 diabetes, Glucose intolerance	[340, 341]
**Plozalizumab**	CCR2	Phase II	Diabetic nephropathy, Melanoma	[342]
**LY-3,041,658**	CXCR1 and CXCR2	Phase II	Hidradenitis suppurativa	[343, 344]
**Avdoralimab**	C5aR1	Phase II	Advanced Solid Tumors, COVID-19 Pneumonia,Herpetic pemphigoid	[345]
**Volagidemab**	GCGR	Phase II	Type 1 diabetes, Type 2 diabetes, Glucose intolerance	[340, 341]
**AMG-301**	PAC1R	Phase II	Migraine	[346]
**Getagozumab**	ETAR	Phase II	Pulmonary arterial hypertension	[347, 348]
**Tidutamab**	SSTR2	Phase II	Merkel cell carcinoma, Small Cell Lung Cancer, Gastrointestinal Mesenchymal Tumor, Neuroendocrine Tumor	[349]
**Nimacimab**	CB1	Phase II	Diabetic gastroparesis, Diabetic nephropathy, Nonalcoholic steatohepatitis, Obesity	[350]
**Ulocuplumab**	CXCR4	Phase II	Pancreatic Cancer, Multiple Myeloma, Acute Myeloid Leukemia, Macroglobulinemia	[351, 352]
**LM-305**	GPRC5D	Phase I/II	Multiple Myeloma, Solid Tumors, Hematologic Diseases	[353]
**TAK-500**	CCR2	Phase I	Breast Cancer, Esophageal Cancer, Nasopharyngeal Cancer, Solid tumor, Head and Neck Squamous Cell Carcinoma, Gastric cancer	[354]
**JBH492**	CCR7	Phase I	B-cell chronic lymphocytic leukemia, Non-Hodgkin’s Lymphoma	[355, 356]
**HZ-515H7**	CXCR4	Phase I	Neoplasms	[357, 358]
**SAR-113,244**	CXCR5	Phase I	Systemic lupus erythematosus	[359]

## Future opportunities and summary

The frontier of GPCR investigation is particularly in the realms of biased agonism, allosteric modulation, and compartmentalized signaling [234, 249, 360, 361]. Each of these avenues presents a unique opportunity to deepen our understanding of GPCR functionality. Biased signaling, for instance, allows for the nuanced control of receptor responses, allosteric modulation offers insights into specific receptor activity manipulation, and compartmentalized signaling provides a framework for understanding the spatial and temporal dynamics of GPCR signaling.

As we navigate through this exciting era of GPCR research, artificial intelligence (AI) stands out as an invaluable tool, playing a promising role in elucidating GPCR structures and facilitating the discovery and development of novel drugs [362]. AI computational capabilities allow for the efficient integration and analysis of vast and complex datasets, aiding in the identification of potential therapeutic targets and the optimization of lead compounds for GPCRs [363]. Through AI-driven virtual and experimental screening processes, novel chemotypes and scaffolds are identified, accelerating the pace of drug discovery and bringing us closer to realizing the therapeutic potential of GPCRs.

Collectively, the collaborative efforts of in-depth GPCR research and AI technology are guiding us toward unprecedented breakthroughs in the field. The integration of modern technology marks a transformative period in our pursuit of understanding and effectively targeting GPCRs for therapeutic intervention, heralding a promising future for precision medicine and drug discovery.

## Data Availability

Not applicable.
